# Direct C2–H alkylation of indoles driven by the photochemical activity of halogen-bonded complexes

**DOI:** 10.3762/bjoc.19.42

**Published:** 2023-04-27

**Authors:** Martina Mamone, Giuseppe Gentile, Jacopo Dosso, Maurizio Prato, Giacomo Filippini

**Affiliations:** 1 Department of Chemical and Pharmaceutical Sciences, INSTM UdR Trieste, University of Trieste, via Licio Giorgieri 1, 34127 Trieste, Italy; 2 Centre for Cooperative Research in Biomaterials (CIC BiomaGUNE), Basque Research and Technology Alliance (BRTA), Paseo de Miramón 194, 20014, Donostia San Sebastián, Spain; 3 Basque Fdn Sci, Ikerbasque, 48013 Bilbao, Spain

**Keywords:** alkylation, EDA complex, halogens, indoles, photochemistry

## Abstract

A light-driven metal-free protocol for the synthesis of sulfone-containing indoles under mild conditions is reported. Specifically, the process is driven by the photochemical activity of halogen-bonded complexes formed upon complexation of a sacrificial donor, namely 1,4-diazabicyclo[2.2.2]octane (DABCO), with α-iodosulfones. The reaction provides a variety of densely functionalized products in good yields (up to 96% yield). Mechanistic investigations are reported. These studies provide convincing evidences for the photochemical formation of reactive open-shell species.

## Findings

**Figure 1:**
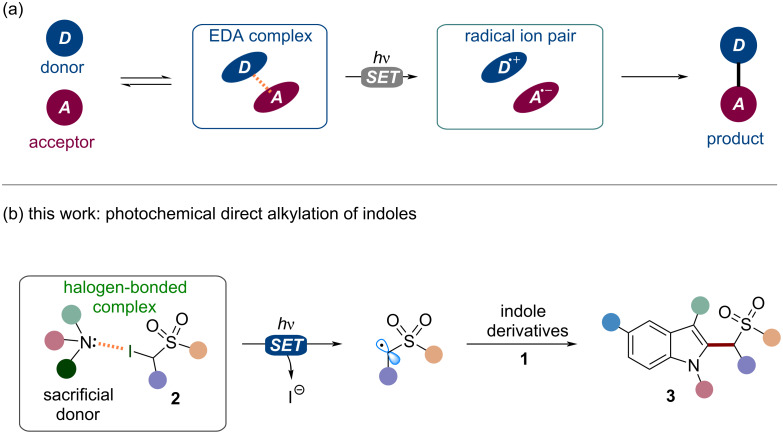
(a) Exploitation of an EDA complex in organic synthesis. (b) This work: use of halogen-bonded complexes to photochemically initiate the C–H alkylation of indoles **1** with iodosulfones **2**.

Direct replacement of carbon–hydrogen (C–H) bonds with new carbon–carbon (C–C) and carbon–heteroatom (C–X) bonds has been and still is a central topic in organic synthesis [1–2]. Historically, organic chemists have extensively relied on the use of noble-metal-based catalysts (e.g., Pd, Rh, Ir, among others) to achieve such type of functionalization [3–5]. However, reliance on noble metal complexes has been constantly declined over recent years due to cost, availability, and toxicity, therefore discouraged by the modern guidelines towards implementation of sustainable chemical production schemes [6]. In the last decades, organic photochemistry has become a prominent tool to guide the development of greener and more convenient synthetic protocols [7–12]. In this context, photochemical approaches based on electron donor–acceptor (EDA) complexes have been successfully exploited to drive the direct C–H functionalization of a large number of organic substrates [13–18]. In this approach, an electron acceptor substrate (“A”) and a donor molecule (“D”) interact to form a new aggregate defined as EDA complex (Figure 1a). Although the two molecular entities might not directly absorb visible light, the newly formed complex usually presents a charge transfer state which results in a bathochromic shift of the absorption towards the visible range [19–20]. Upon light irradiation, the EDA complex may undergo an intramolecular single-electron-transfer (SET) process to produce a radical ion pair (D^•+^, A^•−^). To avoid the occurrence of a back-electron-transfer (BET), a suitable leaving group (LG) needs to be included in one of the precursors. In this manner, reactive intermediates (e.g., radical species) may be generated in solution through the irreversible fragmentation of the substrates [15,21–22]. These intermediates eventually react to yield the final products "A–D". This approach is not limited to reagents with appropriate donor–acceptor characteristics [13,19].

Indeed, sacrificial electron donors and electron-deficient radical precursors can be used to form photoactive EDA complexes. Specifically, these aggregates can be employed to photochemically generate electrophilic radicals that can drive the functionalization of suitable electron-rich substrates [23].

Exploiting this strategy, here we report a novel metal-free methodology for the direct homolytic aromatic substitution (HAS) reaction of indoles **1** with α-iodosulfones **2** to yield the alkylated derivatives **3** (Figure 1b). Indoles play a crucial role in many natural and industrial processes. Therefore, the direct chemical manipulation of the indole system is a matter of paramount importance [24–27]. Moreover, the sulfonyl group is an extremely versatile chemical moiety which may be easily transformed into different functionalities employing conventional synthetic methods. As an example, the sulfonyl group removal under simple reductive treatment may give access to important methylated compounds [12,21]. This operationally simple approach occurs at ambient temperature and under visible-light irradiation. Interestingly, this method employs 1,4-diazabicyclo[2.2.2]octane (DABCO) as sacrificial donor in the EDA complex formation with **2**. To test the feasibility of our design plan, we focused on the reaction between 3-methylindole (**1a**, 2 equiv) and α-iodosulfone **2a** (Table 1).

**Table 1:** Optimization of the reaction conditions and control experiments.

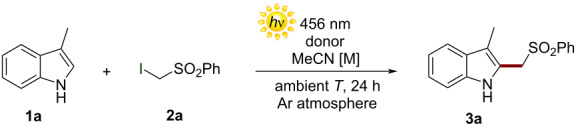					

entry	donor	[M]	**1a**:**2a**:donor (equiv)	light source (nm)	yield (%)^a^

1^b^	DBU	0.5	1:2:1	456	56
2	DBU	0.5	1:2:1	light off	0
3	–	0.5	1:2:1	456	0
4^c^	DBU	0.5	1:2:1	456	0
5^d^	DBU	0.5	1:2:1	456	0
6	2,6-lutidine	0.5	1:2:1	456	0
7	TMG	0.5	1:2:1	456	64
8	NEt_3_	0.5	1:2:1	456	64
9	DABCO	0.5	1:2:1	456	77
10	DABCO	0.25	1:2:1	456	64
11	DABCO	1.0	1:2:1	456	69
12	DABCO	0.5	1:1:1	456	65
13	DABCO	0.5	2:1:1	456	73
14	DABCO	0.5	2:1:1.5	456	95
15^e^	DABCO	0.5	2:1:1.5	456	18
16^f^	DABCO	0.5	2:1:1.5	456	60

^a^Yield determined by ^1^H NMR spectroscopy using 1,3,5-trimethoxybenzene as the internal standard. ^b^Conditions: indole **1a** (0.1 mmol), α-iodosulfone **2a** (0.2 mmol), acetonitrile (MeCN, 200 μL), donor (0.1 mmol), ambient temperature. ^c^Reaction in air. ^d^Reaction performed in the presence of 2 equiv of TEMPO. ^e^Reaction performed in hexane as solvent. ^f^Reaction performed in methanol as solvent.

The experiments were conducted at ambient temperature in acetonitrile (0.5 M) and under irradiation by a Kessil lamp at 456 nm. When adding 1,8-diazabiciclo[5.4.0]undec-7-ene (DBU) as sacrificial donor (1 equiv), the desired product **3a** was formed in good chemical yield (entry 1, Table 1). Control experiments were conducted to obtain more mechanistic clues (entries 2–5, Table 1). An experiment revealed how the exclusion of light completely suppressed the process, therefore establishing the photochemical nature of the transformation (entry 2, Table 1). In addition, we confirmed that DBU was essential for the reactivity, since no reaction occurred in its absence (entry 3, Table 1). Reactivity was also inhibited under an aerobic atmosphere and in the presence of 2,2,6,6‐tetramethylpiperidinyloxyl (TEMPO). These experiments are consonant with the occurrence of a radical mechanism (entries 4 and 5, Table 1) [28]. Afterwards, the effect of the chemical nature of the sacrificial donor on the reaction was investigated (entries 6–9, Table 1). In particular, we employed 2,6-lutidine, 1,1,3,3-tetramethylguanidine (TMG), triethylamine (NEt_3_), and DABCO. Interestingly, the use of DABCO provided the best result in terms of reactivity, yielding compound **3a** in 77% yield. We also observed that either increasing or decreasing the concentration of the reaction mixture did not bring any improvement (entries 10 and 11, Table 1). Then, the ratio between the reagents was optimized. In particular, the use of **2a**:**1a** in a 1:1 ratio resulted in the formation of **3a** in 65% yield (entry 12, Table 1). Moreover, we found that employing an excess of **1a** (2 equiv) led to the formation of **3a** in a 73% yield (entry 13, Table 1). Due to an easier purification of product **3a** from the reaction crude by flash column chromatography, we decided to keep optimizing the transformation using the stoichiometric ratio indicated in entry 13 of Table 1. Importantly, product **3a** was obtained in excellent yield (95%) using 1.5 equivalents of DABCO (entry 14, Table 1). In addition, the use of hexane as solvent provided the desired product **3a** in low chemical yield (entry 15, Table 1). On the other hand, **3a** was obtained in moderate yield (60%) using methanol as solvent (entry 16, Table 1). To shed light on the reaction mechanism, the formation of an EDA complex between the α-iodosulfone **2a** and DABCO was investigated using both UV–vis and nuclear magnetic resonance (NMR) spectroscopy [29].

**Figure 2:**
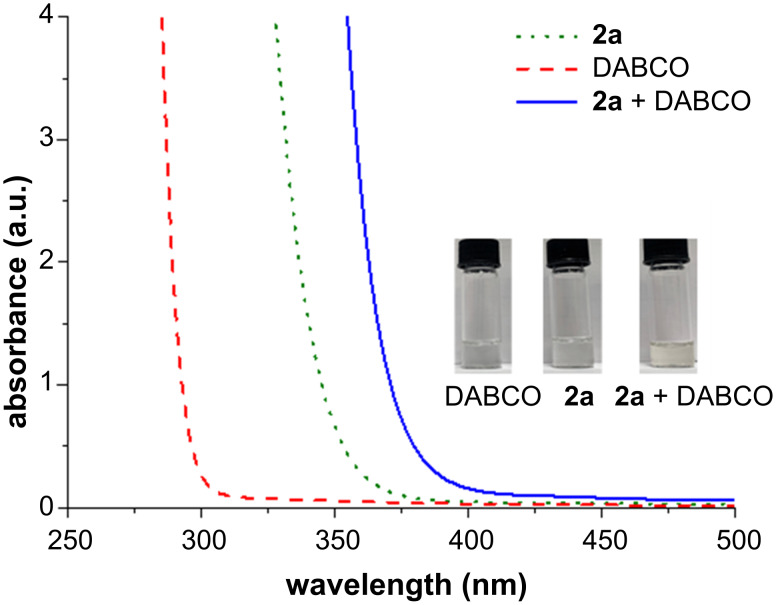
Optical absorption spectra recorded in acetonitrile in 1 cm path quartz cuvettes. [DABCO]: 0.5 M; [**2a**]: 0.5 M.

**Figure 3:**
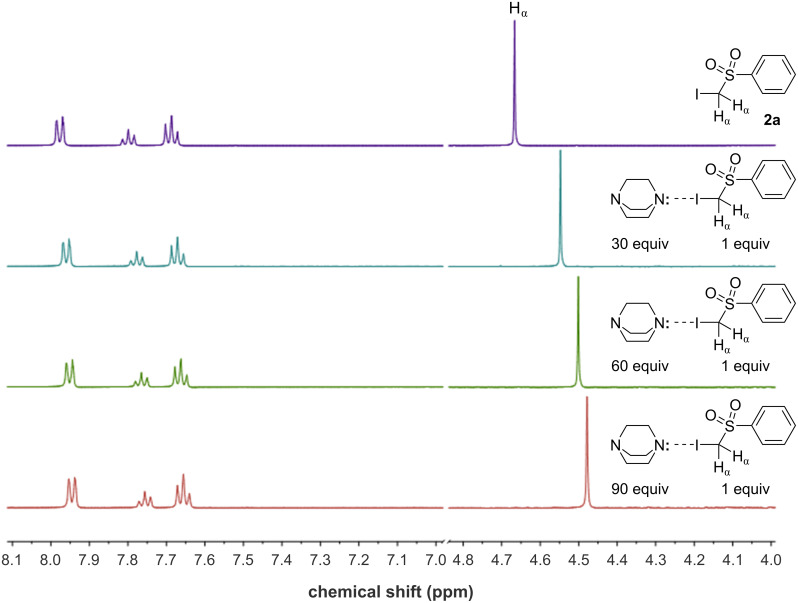
^1^H NMR titration of DABCO in a solution of **2a** in ACN-*d*_3_ to detect their halogen-bonding association through the shift of the signal of H_α_.

In particular, the optical absorption spectra of substrate **2a** (green dotted line), DABCO (red dotted line), and the solution containing both **2a** and DABCO (blue line) were recorded in acetonitrile (Figure 2). Specifically, it was observed that the addition of DABCO to the solution of **2a** induced a bathochromic shift of the absorption spectrum towards the visible region, thus indicating the formation of an EDA complex between these chemical species. Importantly, we also confirmed that indole **1a** and **2a** do not form a photoactive EDA complex when mixed in solution (see Figure S1 in Supporting Information File 1). To further corroborate the hypothesis of an EDA complex being at the roots of the observed reactivity, NMR studies were also performed on samples containing the α-iodosufone **2a** and different concentrations of DABCO in deuterated acetonitrile (Figure 3).

**Figure 4:**
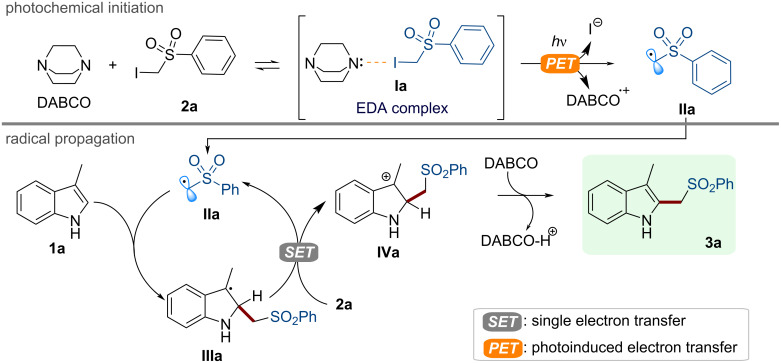
Proposed reaction mechanism for the photochemical alkylation of **1a** with the α-iodosulfone **2a** in the presence of DABCO.

Interestingly, a change in chemical shift of the diagnostic α-protons of **2a** was displayed upon addition of increasing amounts of DABCO, suggesting the presence of the halogen-bonding interaction [30]. More precisely, the ^1^H NMR signal of the α-hydrogens (H_α_) within **2a** was found to shift to lower ppm values because the H_α_ nuclei have been affected by higher electron density caused by the formation of the halogen-bonded complex between **2a** and DABCO. To confirm that the shift of H_α_ was indeed produced by a halogen-bonding interaction, ^19^F NMR analysis of compound **2d**, which presents a difluoromethylene group (–CF_2_–) in the alpha position to the iodine, was performed (see Figure S3 in Supporting Information File 1). Even in this case, an important shift of the fluorine signal was observed. Thus, from a mechanistic point of view, the reaction is driven by the formation of a halogen-bonded EDA complex (**Ia**) between the sulfone **2a** and DABCO (Figure 4).

**Scheme 1:**
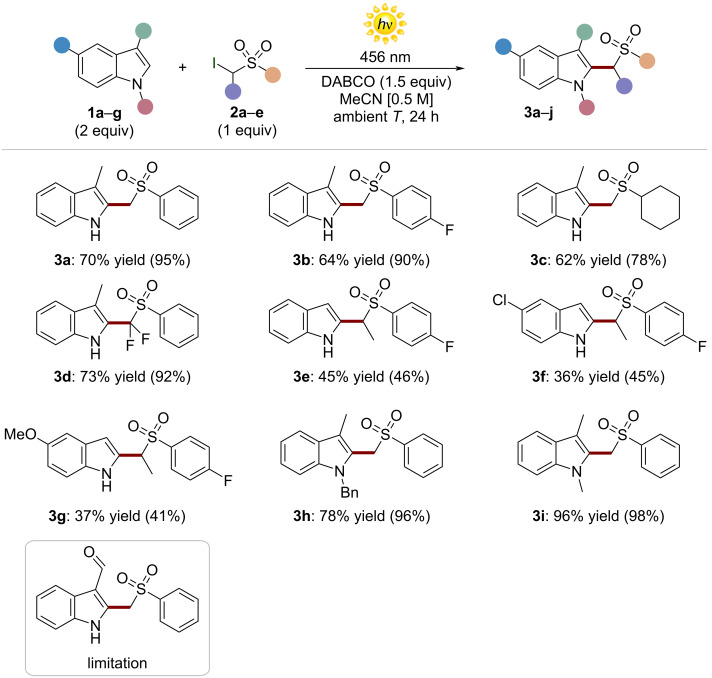
Study of scope of the HAS reaction between indoles **1** and α-iodosulfones **2**. Yields in parentheses were determined by ^1^H NMR analyses, using 1,3,5-trimethoxybenzene as an internal standard.

When irradiated, this photoactive aggregate led to the formation of reactive alkyl radicals (**IIa**), which may react with indole **1a** eventually yielding the product **3a** through a classical HAS pathway [31–33]. Then, we demonstrated the synthetic potential of our photochemical method (Scheme 1).

The reaction could satisfactorily tolerate a diverse set of α-iodosulfones **2** to deliver the corresponding products **3a**–**d** from moderate to excellent yields (up to 73% yield). We found that different indoles actively participated in the photochemical alkylation, leading to the products **3e**–**i** (up to 96% yield). It is worth noting that derivatives **3e**–**g** were isolated in moderate yields as single regioisomer since the alkylation step took place exclusively in position 2 of the starting indoles. As limitation, we observed that indole-3-carboxaldehyde (**1g**) was not a suitable substrate for this transformation.

## Conclusion

In conclusion, we reported a novel photochemical method for the direct C–H alkylation of indoles with α-iodosulfones. This approach exploits the photochemical activity of halogen-bonded EDA complexes, formed between α-iodosulfones and DABCO, that are able to produce reactive *C*-centered radicals under mild reaction conditions.

## Supporting Information

File 1 General procedures and products characterization.
