# Supplementary material for: Direct C2–H alkylation of indoles driven by the photochemical activity of halogen-bonded complexes
Source: Beilstein J Org Chem. 2023 Apr 27;19:575–81. doi: 10.3762/bjoc.19.42 (PMC10155616; doi:10.3762/bjoc.19.42)
Supplement: File 1 — General procedures and products characterization. [file Beilstein_J_Org_Chem-19-575-s001.pdf]

BEILSTEIN JOURNAL OF ORGANIC CHEMISTRY

Supporting Information

for

Direct C2–H alkylation of indoles driven by the photochemical activity of halogen-bonded complexes

Martina Mamone, Giuseppe Gentile, Jacopo Dosso, Maurizio Prato and Giacomo Filippini

Beilstein J. Org. Chem. **2023,** *19,* 575–581. doi:10.3762/bjoc.19.42

General procedures and products characterization

License and Terms: This is a supporting information file under the terms of the Creative Commons Attribution License (https://creativecommons.org/licenses/by/4.0). Please note that the reuse, redistribution and reproduction in particular requires that the author(s) and source are credited and that individual graphics may be subject to special legal provisions.
The license is subject to the *Beilstein Journal of Organic Chemistry* terms and conditions: (https://www.beilstein-journals.org/bjoc/terms)

Table of contents

1. General information S1
2. General procedures for the synthesis of starting materials S2
   2.1 Procedure for the synthesis of indoles **1d** and **1e** S2
   2.2 Procedure for the synthesis of α-iodosulfones **2a**–**e** S3
3. General procedure for the C–H alkylation of indoles S5
4. Absorption spectra S8
5. NMR titration S9
6. NMR spectra S11
7. References S24

1. General information

UV–vis measurements were carried out on a Cary 5000 UV–vis–NIR. All the spectra were recorded at room temperature using 10 mm path-length quartz cuvettes. The NMR spectra were recorded on a Varian 400 spectrometer (^{1}H: 400 MHz; ^{19}F: 376.0 MHz; ^{13}C: 101.0 MHz) or a Varian Inova spectrometer (^{1}H: 500 MHz; ^{13}C: 126 MHz). The chemical shifts (δ) for ^{1}H and ^{13}C NMR spectra are given in ppm relative to residual signals of the solvents ($CHCl_3$ @ 7.26 ppm for ^{1}H NMR, and @ 77.16 ppm for ^{13}C NMR). ATR-IR measurements were performed using a Spectrum 2000 FT-IR Instrument (Perkin Elmer).

General procedures. Chromatographic purification of products was accomplished using flash chromatography on silica gel (SiO_2, 0.04–0.063 mm, 60 Å) or using a Biotage Isolera automated flash chromatography system with cartridges packed with silica (SiO_2, 0.04–0.063 mm, 60 Å). For thin layer chromatography (TLC) analysis throughout this work, Merck precoated TLC plates (silica gel 60 GF254, 0.25 mm) were employed, using UV light as the visualizing agent (254 nm), basic aqueous potassium permanganate ($KMnO_4$) or vanillin stain solutions, and heat as developing agents. Organic solutions were concentrated under reduced pressure on a Büchi rotatory evaporator.

The photochemical homolytic-aromatic-substitution (HAS) reactions between indoles and iodosulfones were set up under an argon atmosphere in Schlenk tubes unless otherwise stated. The light sources used in this work were purchased from Kessil. Details for Kessil lamp: 456 nm PR160L-456 (50 W).

ESI–High resolution mass spectrometry (ESI–HRMS). ESI–HRMS was performed at the University of Trieste Chemistry department, high resolution mass spectra (HRMS) were obtained on a Bruker micrOTOF-Q (ESI–TOF). ***Photophysical analysis***: Absorption spectra of compounds were recorded on air-equilibrated solutions at room temperature with an Agilent Cary 5000 UV–vis spectrophotometer, using quartz cells with a path length of 1.0 cm.

Materials. Commercial reagents and solvents were purchased from Sigma-Aldrich, Fluka, Alfa Aesar, Fluorochem, and VWR. They were used as received, without further purification, unless otherwise stated. Synthesis grade and anhydrous solvents were used as purchased. The preparation of starting materials **1e**,**f**, **2a**–**e** is detailed in Section 2.

2. General procedures for the synthesis of starting materials

2.1 Procedure for the synthesis of indoles 1e and 1f

The synthesis of indoles **1e** and **1f** have been carried out following modified literature procedures [1,2].

Procedure for the synthesis of 1e

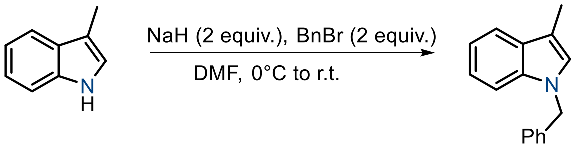

1a

2 mmol
(1 equiv.)

1e

Yield 78%

Procedure for the synthesis of **1e** has been carried out following a modified literature procedure [2]. To a stirred solution of NaH (160 mg, 4 mmol, 60% suspension in mineral oil) in dry DMF (1.6 mL), 3-methylindole (263 mg, 2 mmol, 1.0 equiv) in DMF (1.0 mL) was added dropwise at 0 °C. The mixture was then stirred at room temperature for 30 min. After cooling to 0 °C, a solution of benzyl bromide (475 µL, 4 mmol) in DMF (2.0 mL) was added dropwise. The reaction mixture was stirred at room temperature for another 5 h and then it was quenched by the addition of water and was extracted with ethyl acetate (3 × 10 mL). The combined organic layer was washed with brine, dried over anhydrous Na_2SO_4, and concentrated under reduced pressure. The residue was purified by column chromatography on silica gel (eluent: cyclohexane/CH_2Cl_2 7:3) to give the corresponding product as white solid (345 mg, 78% yield).

^{1}H NMR (400 MHz, $CDCl_3$) δ 7.89 – 7.76 (m, 1H), 7.52 – 7.30 (m, 6H), 7.27 (m, 2H), 7.03 (d, *J* = 0.8 Hz, 1H), 5.36 (s, 2H), 2.55 (d, *J* = 1.0 Hz, 3H). The characterization of the compound matches with the data reported in the literature [2].

Procedure for the synthesis of 1f

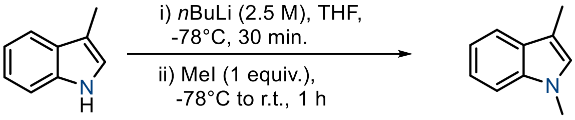

1a

3.8 mmol
(1 equiv.)

1f

Yield 63%

3-Methylindole (3.8 mmol, 500 mg) was added in a flame-dried Schlenk tube under argon and dissolved in anhydrous THF (10 mL). The solution was then cooled to −78 °C (acetone liq. N_2) and *n*-BuLi (2.5 M hexanes) was then added dropwise. After 30 min, the reaction was quenched by the addition of MeI (237 µL, 3.8 mmol), allowed to reach room temperature, and stirred for 1 h at the same temperature. To the resulting solution was then added H_2O (30 mL) and extracted with CH_2Cl_2 (3 × 20 mL). The organic layers were then washed with H_2O (2 × 20 mL) and brine (30 mL), dried over Na_2SO_4, filtered, and evaporated. The residue was purified on a silica gel plug (eluent: PE to PE/CH_2Cl_2 4:1) to give **1f** as a white solid (350 mg, 63% yield).

^{1}H NMR ($CDCl_3$, 400 MHz) δ 7.57 (dt, *J* = 7.9 Hz, 1.0 Hz, 1H), 7.28 (dt, *J* = 8.2 Hz, 0.9 Hz, 1H), 7.22 (ddd, *J* = 8.2 Hz, 6.9 Hz, 1.2 Hz, 1H), 7.11 (ddd, *J* = 7.9 Hz, 6.9 Hz, 1.1 Hz, 1H), 6.83 (d, *J* = 1.0 Hz, 1H), 3.74 (s, 3H), 2.33 (d, *J* = 1.1 Hz, 3H). The characterization of the compound matches with the data reported in the literature [1].

2.2 Procedure for the synthesis of α-iodosulfones 2a–e

Procedure for the synthesis of α-iodosulfones 2a–c

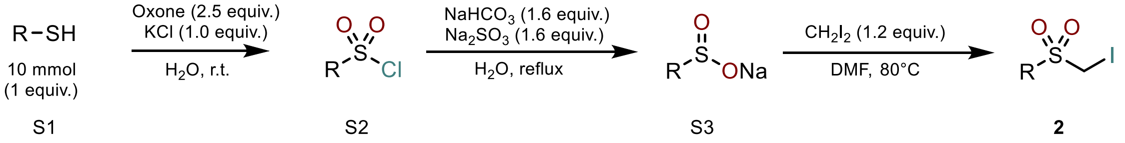

Step 1, has been carried out following a modified literature procedure [3]. A mixture of thiol S1 (10 mmol, 1 equiv), potassium peroxymonosulfate (25 mmol, 2.5 equiv), KCl (10 mmol, 1 equiv), and H_2O (30 mL) were introduced in a single-necked round-bottomed flask and stirred at room temperature for 2 h. The aqueous phase was extracted with ethyl acetate (3 × 50 mL). The combined organic layer was dried over anhydrous Na_2SO_4 and concentrated under reduced pressure. The crude product was purified by flash column chromatography (cyclohexane) affording the desired products S2. The characterization data matched with the reported one [4].

Step 2, according to a literature procedure [5]. The selected sulfonyl chloride S2 (7 mmol, 1 equiv) was dissolved in H_2O (25 mL). Sodium sulfite (11.2 mmol, 1.6 equiv) and sodium bicarbonate (11.2 mmol, 1.6 equiv) were added, and the reaction mixture was refluxed for 3 h. Water was removed by evaporation. Ethanol was added to the solid and the so obtained suspension was heated for 10 min, cooled, and filtered. This procedure was repeated twice using the residue of the filtration. The ethanol fractions were combined, and the solvent was evaporated under reduced pressure. Sodium sulfinate S3 was used without any further purification. The characterization data matched the reported one [4].

Step 3, according to a reported procedure [6]. A solution of sodium sulfinate S3 (5 mmol, 1 equiv) in DMF (20 mL) was stirred at room temperature for 15 min. Diiodomethane (6 mmol, 1.2 equiv) was added dropwise, and the solution was heated at 80 °C for 17 h. The reaction was quenched by the addition of 5% LiCl water solution (100 mL). Subsequently, the aqueous solution was extracted with ethyl acetate (3 × 30 mL). The organic phases were combined and washed with brine (50 mL), saturated solution of sodium thiosulfate (50 mL) and then dried over Na_2SO_4 before being concentred in vacuo. The product was purified by flash column chromatography (eluent: cyclohexane/ethyl acetate) to afford the desired α-iodosulfones **2a**–**c**. (R = -phenyl 88% yield; R = 4-fluorophenyl 75% yield; R = cyclohexyl-35% yield).

Procedure for the synthesis of 2d

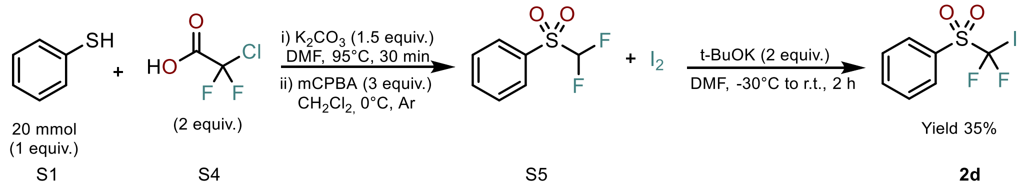

Step 1, was performed following a modified reported procedure [7]. Into an oven-dried schlenk tube equipped with a stirring bar, thiophenol (20 mmol, 1 equiv, 2.2 g) and K_2CO_3 (1.5 equiv, 30 mmol) were introduced and dissolved in dry DMF (25 mL). Chlorodifluoroacetic acid S4 (2.0 equiv, 40 mmol) was solubilized in dry DMF and added dropwise into the reaction mixture. The reaction was kept under argon atmosphere. The reaction was then heated at 95 °C for 30 min. Subsequently, it was quenched through the addition of 5% LiCl water solution and extracted with cyclohexane (3 × 20 mL). The organic layers were reunited, dried with Na_2SO_4, and concentrated under reduced pressure. The crude intermediate from the first step was used as obtained after extraction. It was dissolved in 20 mL of CH_2Cl_2 under argon and cooled to 0 °C. m-Chloroperoxybenzoic acid (60 mmol, 3 equiv) was slowly added. The reaction was stirred overnight (approximately 16 h). The day after, 0.1 M NH_4Cl water was added and the product S5 was extracted with ethyl acetate (3 × 20mL). The ethyl acetate fractions were combined and dried over Na_2SO_4 and the solvent was evaporated under reduced pressure. Product S5 was obtained with a yield of 40%.

^{1}H NMR ($CDCl_3$, 400 MHz) δ 6.20 (t, J = 53.1 Hz, 1H), 7.62-7.68 (m, 2H), 7.79-7.85 (m, 1H), 7.97-8.02 (m, 2H). **^{19}F NMR ($CDCl_3$, 376 MHz)** δ -121.9 (d, J = 53.5 Hz, 2F). The characterization of the compound matches with the data reported in the literature [8].

Step 2, The last reaction step was carried out according to the literature [9]. S5 (8.5 mmol, 1.470 g) was introduced in an oven-dried double-necked round-bottomed flask with a stirring bar along with dry DMF (20 mL), cooled to −30 °C and placed under argon. I_2 (2.5 equiv, 21.25 mmol) was added in small aliquots followed by a dropwise addition of *t*-BuOK (2.0 equiv, 17 mmol). The reaction mixture was stirred at −30 °C for 1 h. Subsequently, the reaction mixture was allowed to reach room temperature in approximately 1 h. A saturated NaCl aqueous solution (15 mL) was added, and the mixture was extracted with Et_2O (3 × 15 mL). The combined organic phase was dried over Na_2SO_4, and the solvent was removed in a rotary evaporator. The crude was purified by silica gel column chromatography (eluent: cyclohexane/ethyl acetate 9:1) to afford the product **2d** (2.22 g, 35% overall yield) as a white crystalline solid. The product has been kept in the dark at 4 °C.

^{1}H NMR (400 MHz, $CDCl_3$) δ 7.65 (t, J = 7.3 Hz, 2H); 7.82 (t, J = 7.4 Hz, 1H); 7.99 (d, J = 7.5 Hz, 2H). **^{19}F NMR (376 MHz, $CDCl_3$)** δ – 51.9 (s, 2F). The characterization of the compound matches with the data reported in the literature [8].

Procedure for the synthesis of 2e

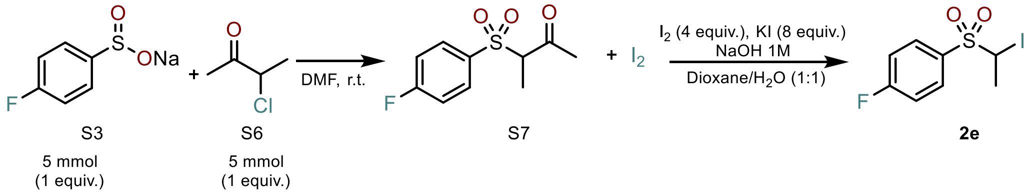

Step 1, according to a modified literature procedure [6]. S3 (5 mmol, 1 equiv) and 3-chloro butanone S6 (5 mmol, 1 equiv) were mixed in DMF (10 mL, 0.5 M). The reaction mixture was stirred at room temperature for 24 h. The reaction was quenched by the addition of water (50 mL) and extracted with ethyl acetate (3 × 35 mL), dried over Na_2SO_4, and the solvent was removed under reduced pressure. The corresponding adduct S7 was used without any further purification.

Step 2, iodine (10 mmol, 4 equiv) was added to a dioxane/water 1:1 (0.5 M) solution of the starting material S7 (2.5 mmol, 1 equiv) in the presence of potassium iodide (20 mmol, 8 equiv). Then, a 1 M solution of NaOH was added dropwise until the discolouration of the excess of iodine. After 20 min of stirring, the mixture was diluted with water and extracted with CH_2Cl_2 (3 × 20 mL). The final α-iodosulfone **2e** was used without any further purification.

3. General procedure for the C-alkylation of indoles

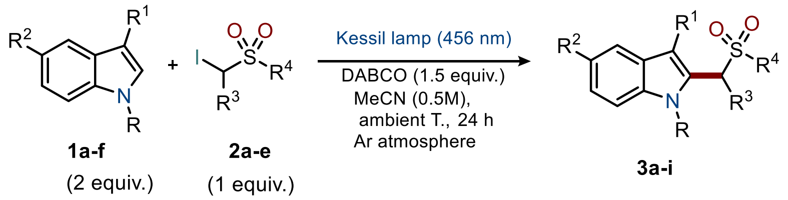

A 10 mL Schlenk tube was charged with indole **1** (0.2 mmol), α-iodosulfone 2 (0.1 mmol, 1.0 equiv), and DABCO (0.15 mmol, 1.5 equiv). Acetonitrile was then added to this mixture (200 μL). The reaction mixture was thoroughly degassed via 3 cycles of freeze-pump-thaw, and the vessel was refilled with argon and placed under light irradiation for 24 h (λ = 456 nm). The temperature was kept at around 30 °C by using a fan. The reaction was then quenched with an aqueous solution of HCl (5 mL, 0.5 M). The mixture was extracted with ethyl acetate (3 × 10 mL). The volatiles were removed in vacuo and the residue was purified by column chromatography (eluent: cyclohexane/CH_2Cl_2) to give the C–H functionalization products **3**.

Characterization data for compounds 3

3-Methyl-2-((phenylsulfonyl)methyl)-1*H*-indole (3a)

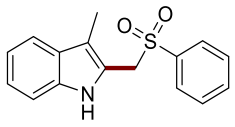

3a

Prepared according to the general procedure 3 using indole **1a** (0.2 mmol, 26 mg), and α-iodosolfone **2a** (0.1 mmol, 28 mg). The product **3a** was obtained as white solid (20 mg, 70% yield).

^{1}H NMR (499 MHz, CDCl$_3$) δ 8.50 (s, 1H), 7.63 – 7.56 (m, 3H), 7.45 – 7.38 (m, 3H), 7.36 (d, J = 8.2 Hz, 1H), 7.25 – 7.18 (m, 1H), 7.14 – 7.06 (m, 1H), 4.50 (s, 2H), 1.70 (s, 3H). **^{13}C NMR (126 MHz, CDCl$_3$)** δ 137.50, 136.56, 134.10, 129.21, 128.43, 128.13, 123.18, 121.08, 119.60, 119.12, 113.45, 111.23, 54.48, 7.80. The characterization of the compound matches with the data reported in the literature [10].

2-(((4-Fluorophenyl)sulfonyl)methyl)-3-methyl-1*H*-indole (3b)

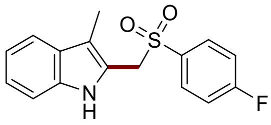

3b

Prepared according to the general procedure 3 using indole **1a** (0.2 mmol, 26 mg), and α-iodosulfone **2b** (0.1 mmol, 30 mg). The product **3b** was obtained as white solid (20 mg, 64% yield).

^{1}H NMR (400 MHz, CDCl$_3$) δ 8.50 (s, 1H), 7.63 – 7.53 (m, 2H), 7.43 (d, *J* = 8.1 Hz, 1H), 7.39 – 7.33 (m, 1H), 7.23 (m, 1H), 7.15 – 7.03 (m, 3H), 4.49 (s, 2H), 1.72 (s, 3H). **^{19}F NMR (376 MHz, CDCl$_3$)** δ -102.73 (tt, *J* = 8.3, 5.1 Hz). **^{13}C NMR (101 MHz, CDCl$_3$)** δ 166.16 (d, *J* = 257.1 Hz), 136.55, 133.44 (d, *J* = 3.2 Hz), 131.36 (d, *J* = 9.7 Hz), 128.06, 123.35, 120.88, 119.74, 119.20, 116.51 (d, *J* = 22.6 Hz), 113.47, 111.24, 54.58, 7.89. **IR (ATR) ν (cm^{-1}):** 3354, 2918, 1589, 1492, 1460, 1336, 1286, 1236, 1170, 1151, 1138, 1083, 875, 839, 761, 740, 725, 707, 692, 651, 626, 584, 528, 511, 482, 432. **HRMS** calculated for $C_{13}H_{14}FNO_2S$ (M-Na): 326.0623, found: 326.0621.

2-((Cyclohexylsulfonyl)methyl)-3-methyl-1*H*-indole (3c)

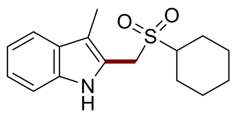

3c

Prepared according to the general procedure 3 using indole **1a** (0.2 mmol, 26 mg), and α-iodosulfone **2c** (0.1 mmol, 29 mg). The product **3c** was obtained as white solid (20 mg, 62% yield).

^{1}H NMR (499 MHz, CDCl$_3$) δ 8.65 (s, 1H), 7.56 (d, *J* = 7.9 Hz, 1H), 7.36 (d, *J* = 8.1 Hz, 1H), 7.27 – 7.21 (m, 1H), 7.18 – 7.10 (m, 1H), 4.40 (s, 2H), 2.72 (tt, *J* = 12.2, 3.5 Hz, 1H), 2.31 (s, 3H), 2.14 – 1.98 (m, 2H), 1.87 (dd, *J* = 7.4, 2.6 Hz, 2H), 1.67 (d, *J* = 5.6 Hz, 1H), 1.60 – 1.45 (m, 2H), 1.25 – 1.05 (m, 3H). **^{13}C NMR (101 MHz, CDCl$_3$)** δ 136.58, 128.35, 123.23, 121.30, 119.75, 119.10, 112.07, 111.37, 59.06, 48.02, 25.11, 25.02, 24.98, 8.82. **IR (ATR) ν (cm^{-1}):** 3401, 2961, 2926, 2849, 1493, 1443, 1396, 1335, 1302, 1288, 1265, 1244, 1167, 1126, 1115, 1098, 876, 854, 772, 743, 719, 696, 650, 584, 546, 530, 513, 496, 430. **HRMS** calculated for $C_{16}H_{21}NO_2S$ (M-Na): 314.1184, found: 314.1185.

2-(Difluoro(phenylsulfonyl)methyl)-3-methyl-1*H*-indole (3d)

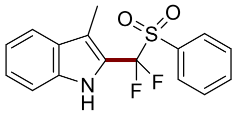

3d

Prepared according to the general procedure 3 using indole **1a** (0.2 mmol, 44 mg), and α-iodosulfone **2d** (0.1 mmol, 28 mg). The product **3d** was obtained as white solid (24 mg, 73% yield).

^{1}H NMR (499 MHz, CDCl$_3$) δ 8.46 (s, 1H), 7.96 (d, *J* = 7.7 Hz, 2H), 7.77 (ddd, *J* = 8.7, 2.4, 1.2 Hz, 1H), 7.65 – 7.58 (m, 3H), 7.39 (d, *J* = 8.3 Hz, 1H), 7.36 – 7.30 (m, 1H), 7.18 (ddd, *J* = 7.0, 5.4, 0.9 Hz, 1H), 2.31 (t, *J* = 2.5 Hz, 3H). **^{19}F NMR (376 MHz, CDCl$_3$)** δ -99.73, -99.73. **^{13}C NMR (126 MHz, CDCl$_3$)** δ 136.69, 135.57, 132.77, 130.92, 129.50, 128.24, 125.32, 120.44, 120.42 (t, J = 285.4 Hz), 120.21, 118.87 (t, J = 2.8 Hz), 117.97 (t, J = 27.2 Hz), 111.82, 8.72 (t, *J* = 2.0 Hz). The characterization of the compound matches with the data reported in the literature [7].

2-(1-((4-Fluorophenyl)sulfonyl)ethyl)-1*H*-indole (3e)

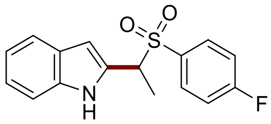

3e

Prepared according to the general procedure 3 using indole **1b** (0.2 mmol, 23 mg), and α-iodosulfone **2e** (0.1 mmol, 31 mg). The product **3e** was obtained as white solid (14 mg, 45% yield).

^{1}H NMR (400 MHz, CDCl$_3$) δ 8.83 (s, 1H), 7.49 (ddd, *J* = 8.0, 5.0, 2.6 Hz, 3H), 7.42 (d, *J* = 8.2 Hz, 1H), 7.23 (dd, *J* = 8.2, 1.1 Hz, 1H), 7.13 – 7.08 (m, 1H), 7.07 – 7.00 (m, 2H), 6.16 – 6.10 (m, 1H), 4.48 (q, *J* = 7.1 Hz, 1H), 1.75 (d, *J* = 7.1 Hz, 3H). **^{19}F NMR**

(376 MHz, CDCl3) δ -102.81 (dd, *J* = 9.2, 4.2 Hz). **^{13}C NMR (101 MHz, CDCl3)** δ 166.14 (d, *J* = 257.0 Hz), 136.94, 132.08 (d, *J* = 9.6 Hz), 130.41, 127.40, 123.23, 120.88, 120.35, 116.29 (d, *J* = 22.7 Hz), 111.37, 110.16, 104.24, 60.48, 13.63. **IR (ATR) ν (cm^{-1}):** 3350, 2918, 2849, 1589, 1491, 1454, 1429, 1404, 1344, 1296, 1285, 1159, 1136, 1096, 1084, 1067, 1047, 1011, 993, 945, 932, 835, 816, 799, 745, 729, 708, 696, 667, 648, 540, 523, 496, 430. **HRMS** calculated for $C_{16}H_{14}FNO_2S$ (M-Na): 326.0621, found: 326.0624.

5-Chloro-2-(1-((4-fluorophenyl)sulfonyl)ethyl)-1*H*-indole (3f)

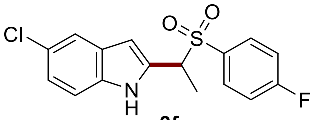

3f

Prepared according to the general procedure 3 using indole **1c** (0.2 mmol, 30 mg), and α-iodosulfone **2e** (0.1 mmol, 31 mg). The product **3f** was obtained as white solid (12 mg, 36% yield).

^{1}H NMR (499 MHz, CDCl3) δ 8.88 (s, 1H), 7.51 – 7.46 (m, 2H), 7.45 (d, *J* = 1.9 Hz, 1H), 7.34 (d, *J* = 8.7 Hz, 1H), 7.19 (dd, *J* = 8.7, 2.0 Hz, 1H), 7.11 – 7.00 (m, 2H), 6.11 – 6.02 (m, 1H), 6.14 – 6.00 (m, 1H), 1.74 (d, *J* = 7.1 Hz, 3H). **^{19}F NMR (376 MHz, CDCl3)** δ -102.43 – -102.53 (m). **^{13}C NMR (126 MHz, CDCl3)** δ 166.05 (d, *J* = 257.4 Hz), 135.23, 132.04 (d, *J* = 9.8 Hz), 131.94, 131.14 – 130.98 (m), 128.40, 125.40, 123.64, 120.25, 116.23 (d, *J* = 22.6 Hz), 112.41, 103.79, 60.36, 13.57. **IR (ATR) ν (cm^{-1}):** 3356, 1589, 1491, 1449, 1310, 1287, 1161, 1138, 1084, 1057, 916, 874, 837, 795, 729, 710, 694, 671, 638, 556, 523, 478, 434, 419. **HRMS** calculated for $C_{16}H_{13}ClFNO_2S$ (M-Na): 360.0235, found: 360.0232.

5-Methoxy-2-(1-((4-fluorophenyl)sulfonyl)ethyl)-1*H*-indole (3g)

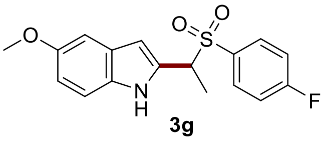

3g

Prepared according to the general procedure 3 using indole **1d** (0.2 mmol, 30 mg), and α-iodosulfone **2e** (0.1 mmol, 31 mg). The product **3g** was obtained as white solid (12 mg, 37% yield).

^{1}H NMR (499 MHz, CDCl3) δ 8.68 (s, 1H), 7.54 – 7.45 (m, 2H), 7.30 (d, *J* = 8.7 Hz, 1H), 7.10 – 7.00 (m, 2H), 6.91 (dt, *J* = 8.7, 2.4 Hz, 2H), 6.05 (d, *J* = 2.1 Hz, 1H), 4.45 (q, *J* = 7.2 Hz, 1H), 3.82 (s, *J* = 7.6 Hz, 3H), 1.74 (d, *J* = 7.1 Hz, 3H). **^{19}F NMR (376 MHz, CDCl3)** δ -102.92, -102.93. **^{13}C NMR (126 MHz, CDCl3)** δ 163.57 (d, *J* = 257.5 Hz), 154.52, 132.08 (d, *J* = 9.6 Hz), 131.44, 130.94, 130.22, 127.87, 116.27 (d, *J* = 22.7 Hz), 113.62, 112.11, 104.00, 102.32, 60.53, 55.90, 13.60. **IR (ATR) ν (cm^{-1}):** 3356, 2916, 2849, 1626, 1589, 1489, 1341, 1312, 1287, 1211, 1175, 1086, 1059, 1032, 943, 808, 787, 762, 743, 708, 671, 638, 619, 561, 527, 467, 432, 419. **HRMS** calculated for $C_{16}H_{16}FNO_3S$ (M-Na): 356.0733, found: 356.0727.

1-Benzyl-3-methyl-2-((phenylsulfonyl)methyl)-1*H*-indole (3h)

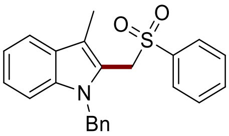

3h

Prepared according to the general procedure 3 using indole **1e** (0.2 mmol, 44 mg), and α-iodosulfone **2a** (0.1 mmol, 28 mg). The product **3h** was obtained as white solid (29 mg, 78% yield).

^{1}H NMR (499 MHz, CDCl3) δ 7.65 (m, 3H), 7.48 (m, 3H), 7.36 – 7.17 (m, 5H), 7.13 (t, *J* = 7.4 Hz, 1H), 6.87 (d, *J* = 6.8 Hz, 2H), 5.56 (s, 2H), 4.41 (s, 2H), 1.66 (s, 3H). **^{13}C NMR (126 MHz, CDCl3)** δ 137.84, 137.65, 137.57, 134.01, 129.14, 128.87, 128.73, 127.52, 127.43, 125.88, 123.04, 122.64, 119.37, 119.16, 114.26, 109.67, 53.38, 46.65, 8.12. **IR (ATR) ν (cm^{-1}):** 3026, 2918, 1651, 1584, 1537, 1497, 1462, 1447, 1250, 1219, 1165,

1138, 1121, 1080, 1024, 999, 885, 799, 773, 748, 731, 714, 696, 681, 646, 611, 544, 525, 517, 500, 455, 424. **HRMS** calculated for $C_{23}H_{21}NO_2S$ (M-Na): 376.1364, found: 376.1366.

1,3-Dimethyl-2-((phenylsulfonyl)methyl)-1*H*-indole (3i)

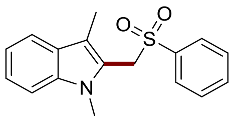

3i

Prepared according to the general procedure 3 using indole **1f** (0.2 mmol, 23 mg), and α-iodosulfone **2a** (0.1 mmol, 31 mg). The product **3i** was obtained as white solid (29 mg, 96% yield).

^{1}H NMR (499 MHz, CDCl$_3$) δ 7.70 – 7.57 (m, 3H), 7.51 – 7.39 (m, 3H), 7.35 – 7.21 (m, 2H), 7.10 (t, J = 7.3 Hz, 1H), 4.56 (s, 2H), 3.72 (s, 3H), 1.66 (s, 3H). **^{13}C NMR (126 MHz, CDCl$_3$)** δ 137.94, 137.68, 134.11, 129.23, 128.88, 127.43, 122.98, 122.83, 119.24, 119.16, 113.24, 109.46, 53.38, 30.38, 8.21. **IR (ATR) ν (cm^{-1}):** 3046, 2936, 1614, 1585, 1470, 1447, 1418, 1385, 1364, 1333, 1315, 1306, 1250, 1182, 1146, 1086, 1070, 1028, 997, 972, 891, 839, 783, 773, 737, 704, 687, 665, 646, 592, 563, 548, 523, 503, 444, 428, 415, 407. **HRMS** calculated for $C_{17}H_{17}NO_2S$ (M-Na): 322.0877, found: 322.0872.

4. Absorption spectra

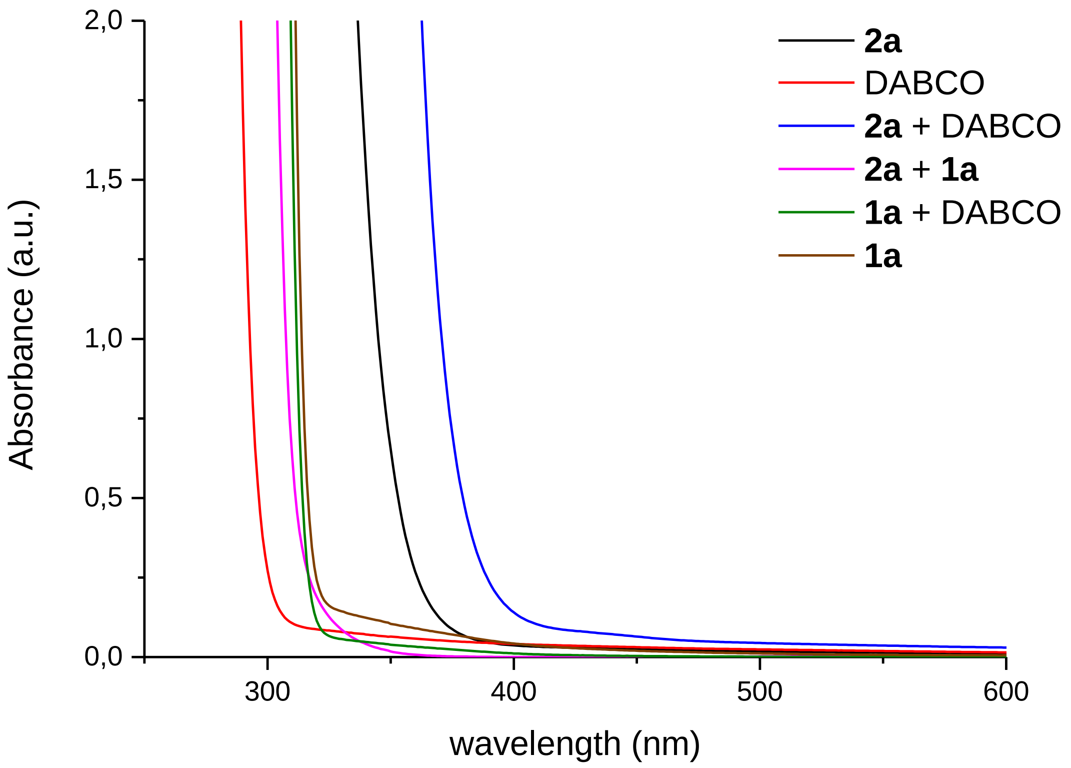

Figure S1: Optical absorption spectra of α-iodosulfone **2a** (black line); DABCO (red line); the mixture between α-iodosulfone **2a** and DABCO (blue line); the mixture between α-iodosulfone **2a** and 3-methylindole (**1a**, violet line); the mixture between DABCO and 3-methylindole (**1a**, green line); 3-methylindole (**1a**, brown line). Spectra were recorded in CH_3CN in quartz cuvettes (1 cm path). [**1a**] = [DABCO] = [**2a**] = 0.05 M.

5. NMR titration

General procedure

A solution of host iodosulfone **2a** (17 mM) in ACN-d_3 was prepared. To this solution, 10 equiv of guest DABCO was added as solid until saturation of the change in chemical shift ($\Delta\delta$) at the host's observed nuclei was reached (100 equiv of DABCO). 1,3,5-Trimethoxybenzene (δ = 6.1 ppm) was used as an internal standard for the determination of DABCO in solution.The constant component during the titration is called "host" and the varied component is called "guest" throughout. ^1H and ^{19}F NMR spectra were recorded on a 500 and 400 MHz Varian Mercury spectrometer.

^1H NMR studies

Regarding the ^1H NMR studies, a change to lower chemical shift of the diagnostic α-protons of **2a** was displayed upon addition of increasing amounts of DABCO, suggesting the presence of the halogen-bonding interaction. After adding 100 equiv of DABCO, the saturation of the formation of the complex was achieved.

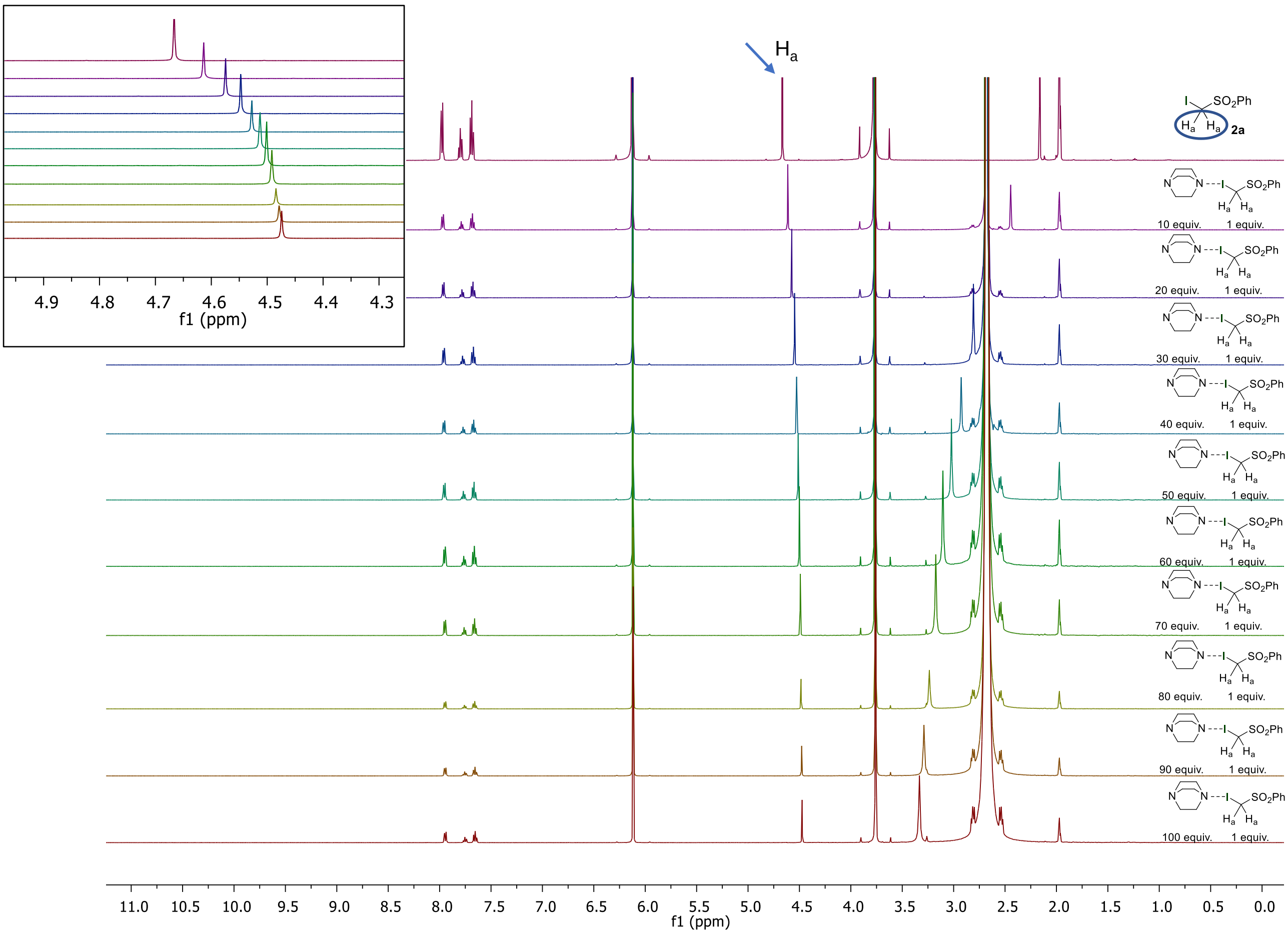

Figure S2: ^1H NMR spectra of titration between host **2a** and increasing quantity of DABCO until 100 equiv.

^{19}F NMR studies

Regarding the ^{19}F NMR studies, a change to lower chemical shift of the diagnostic α-fluorine of **2d** was displayed upon addition of increasing amounts of DABCO, suggesting the presence of the halogen-bonding interaction. An important shift of the fluorine signal was observed confirming that the halogen-bonding interaction between **2a** and DABCO.

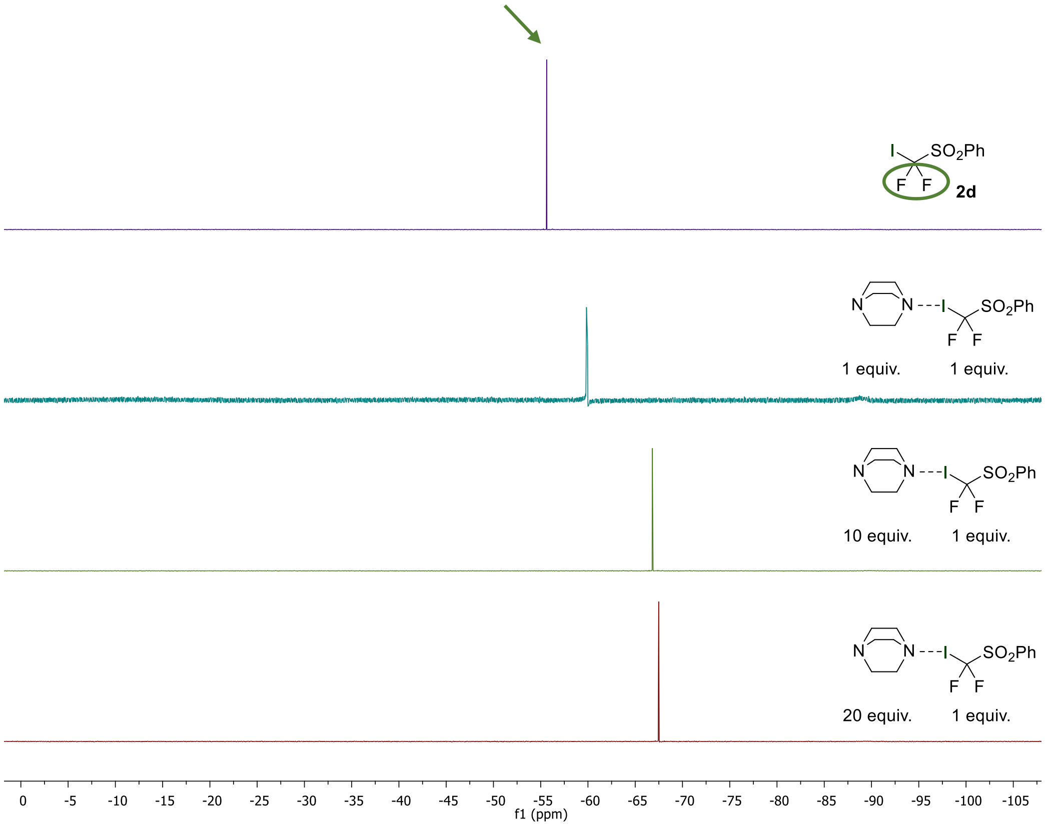

Figure S3: ^{19}F NMR spectra of titration between host **2d** and increasing quantity of DABCO until 20 equiv.

6. NMR spectra

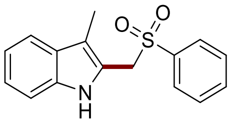

3a

^1H NMR (499 MHz, $CDCl_3$)

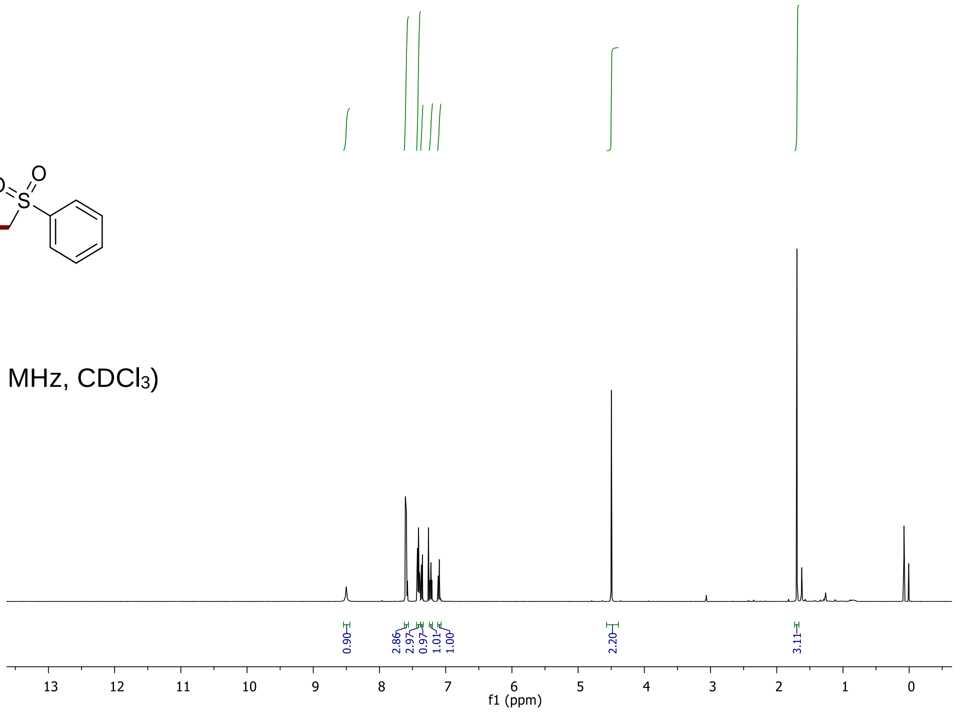

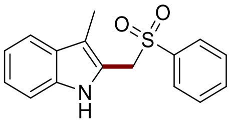

3a

^{13}C NMR (126 MHz, $CDCl_3$)

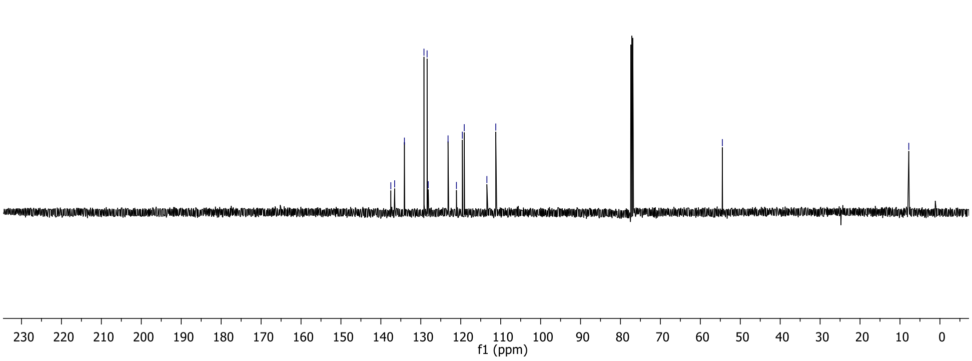

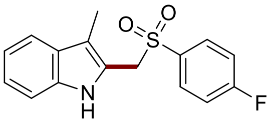

3b

^{1}H NMR (400 MHz, CDCl$_3$)

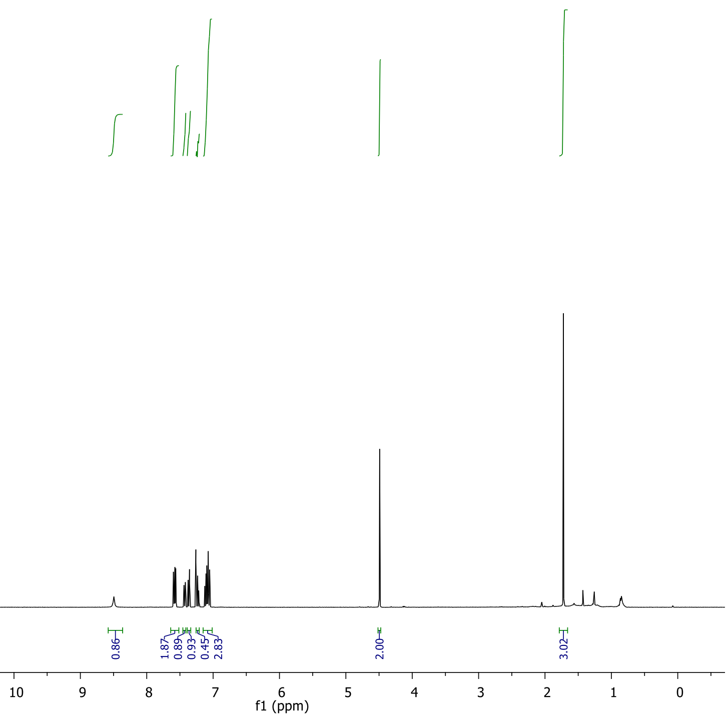

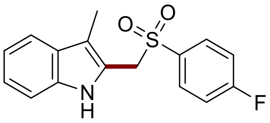

3b

^{19}F NMR (376 MHz, CDCl$_3$)

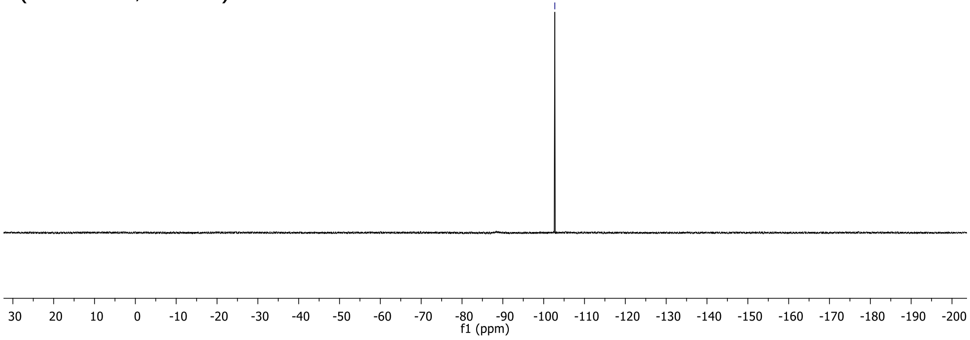

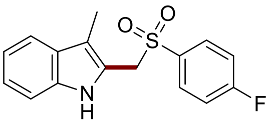

3b

^{13}C NMR (101 MHz, CDCl$_3$)

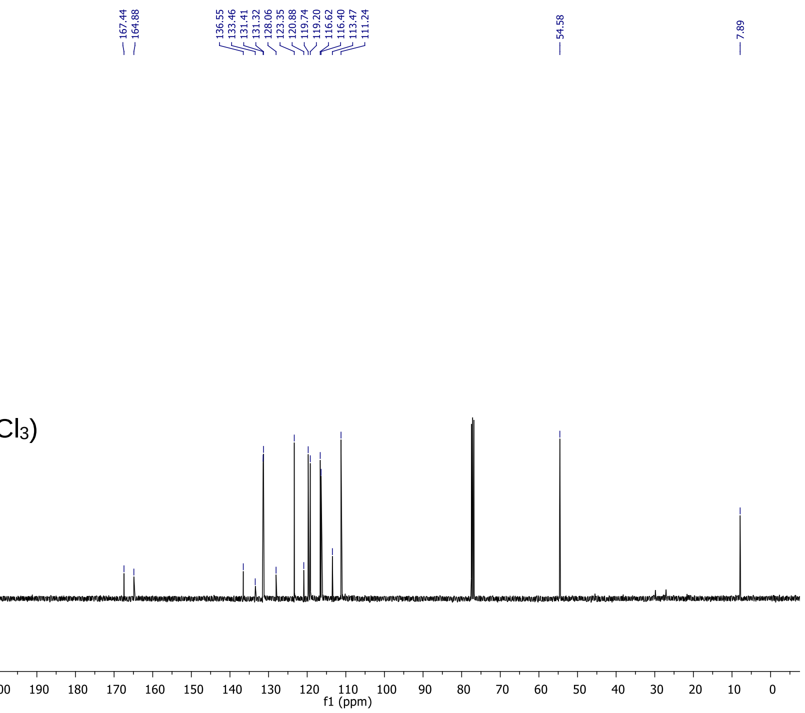

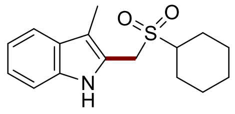

3c

^{1}H NMR (499 MHz, CDCl$_3$)

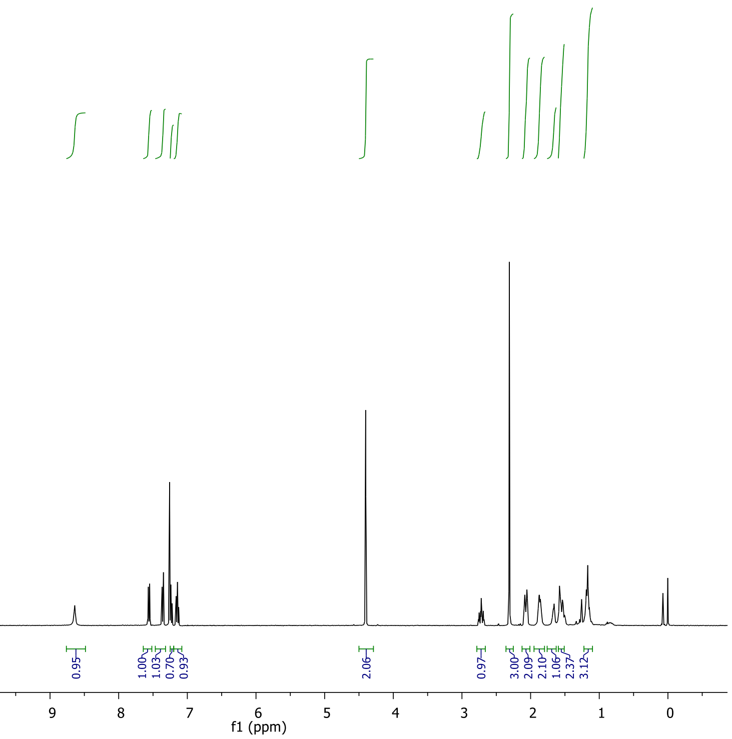

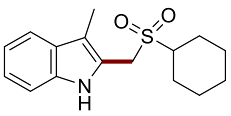

3c

^{13}C NMR (101 MHz, $CDCl_3$)

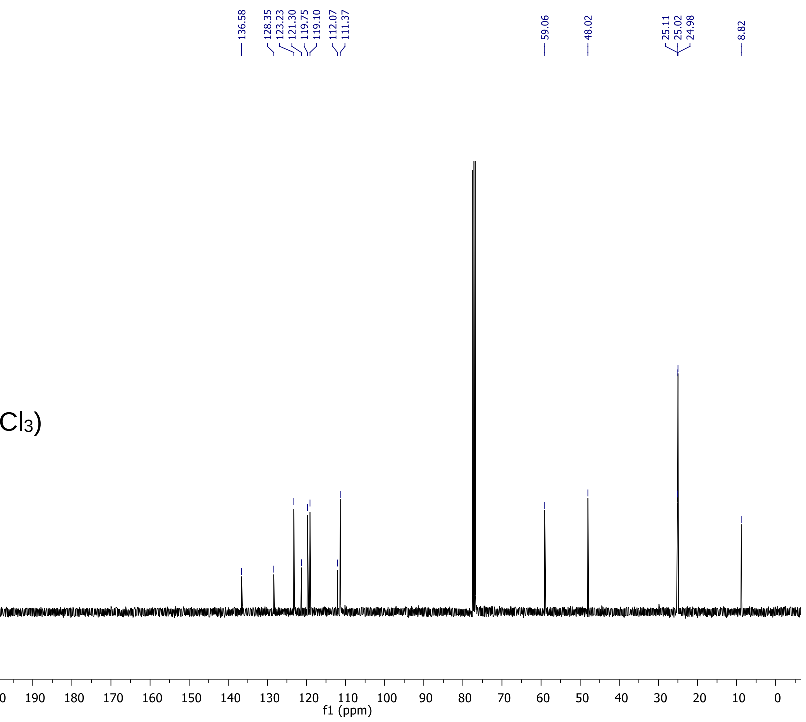

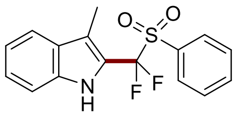

3d

^{1}H NMR (499 MHz, $CDCl_3$)

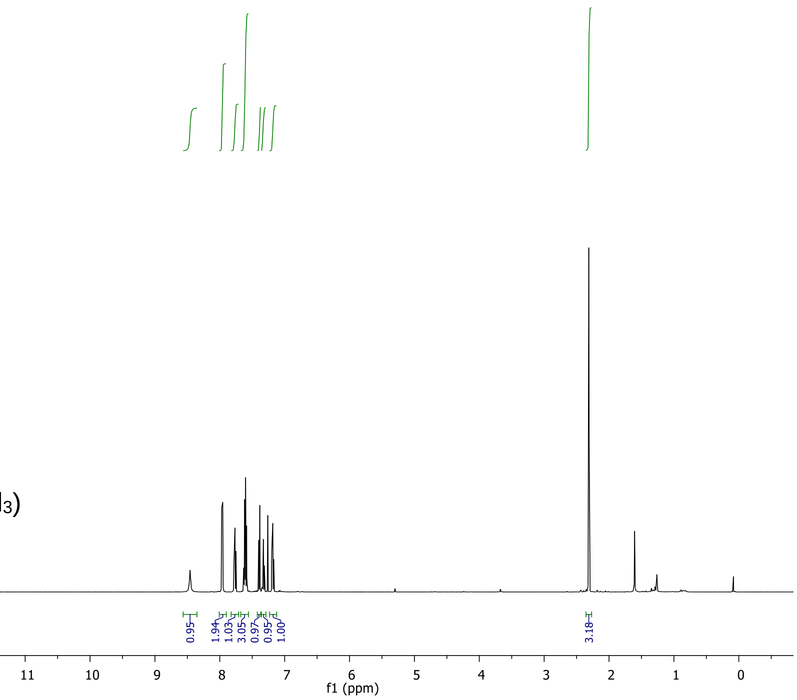

3d

^{19}F NMR (376 MHz, $CDCl_3$)

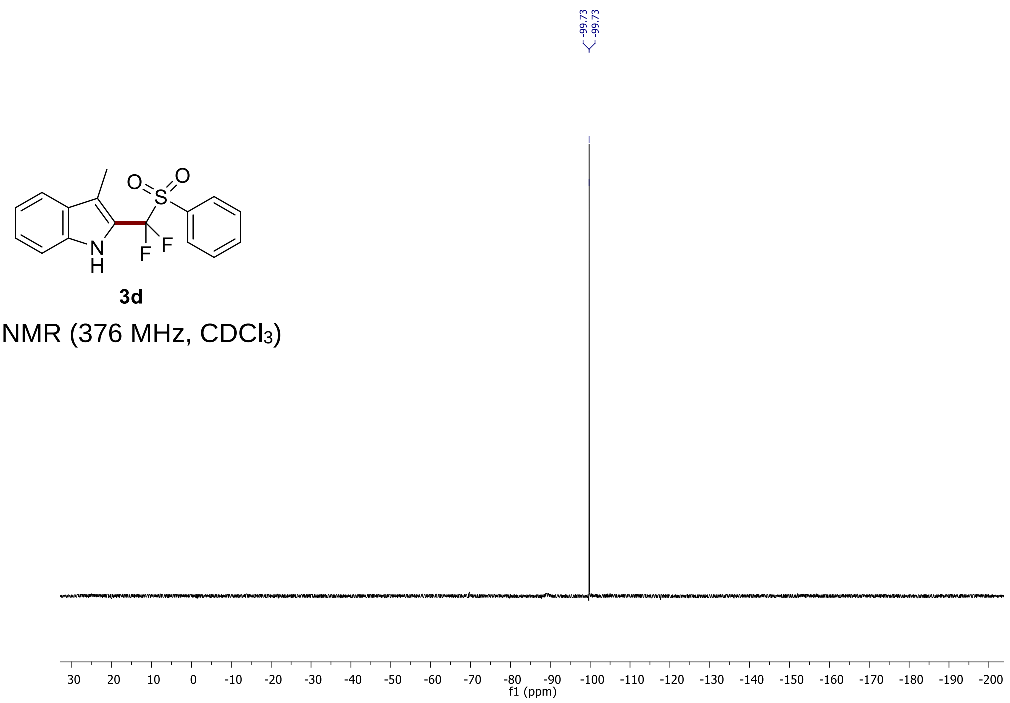

3d

^{13}C NMR (126 MHz, $CDCl_3$)

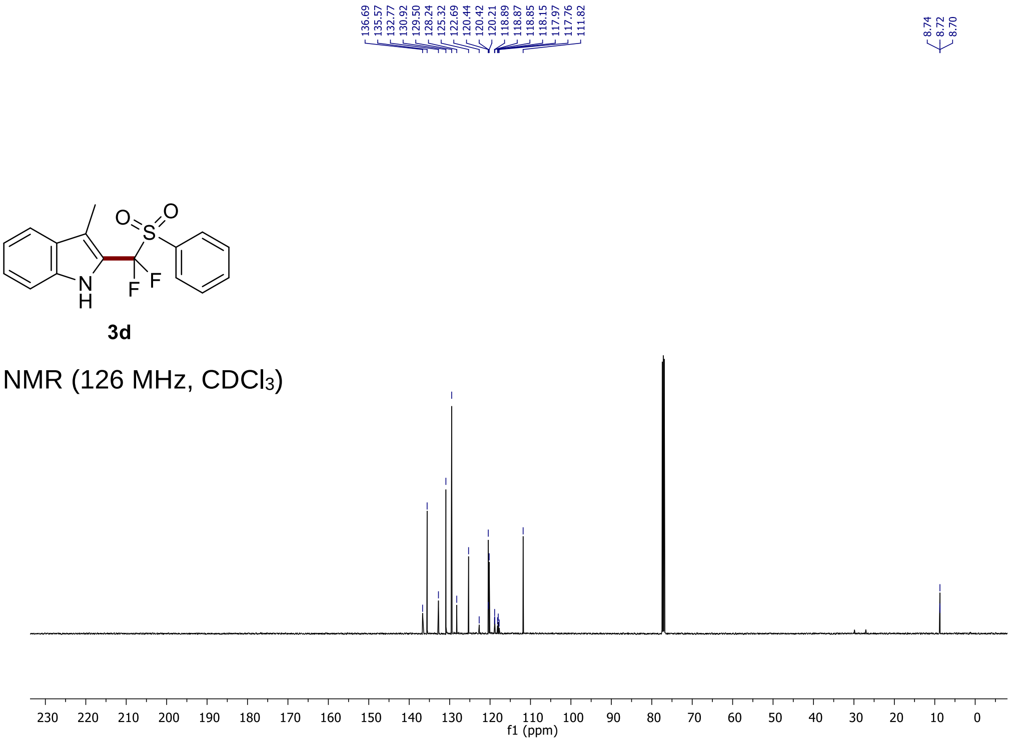

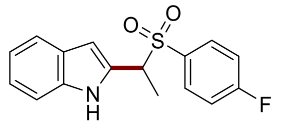

3e

^{1}H NMR (400 MHz, $CDCl_3$)

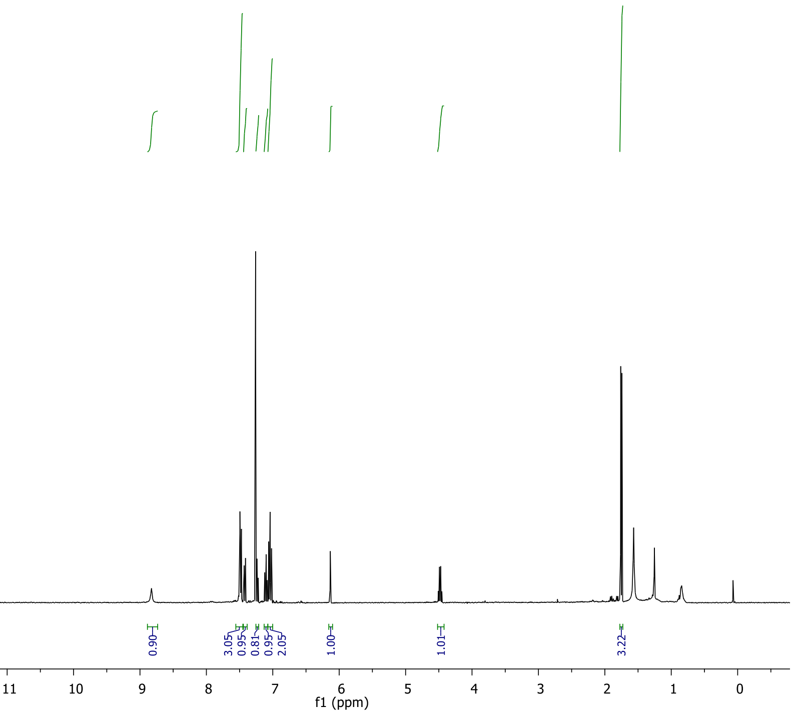

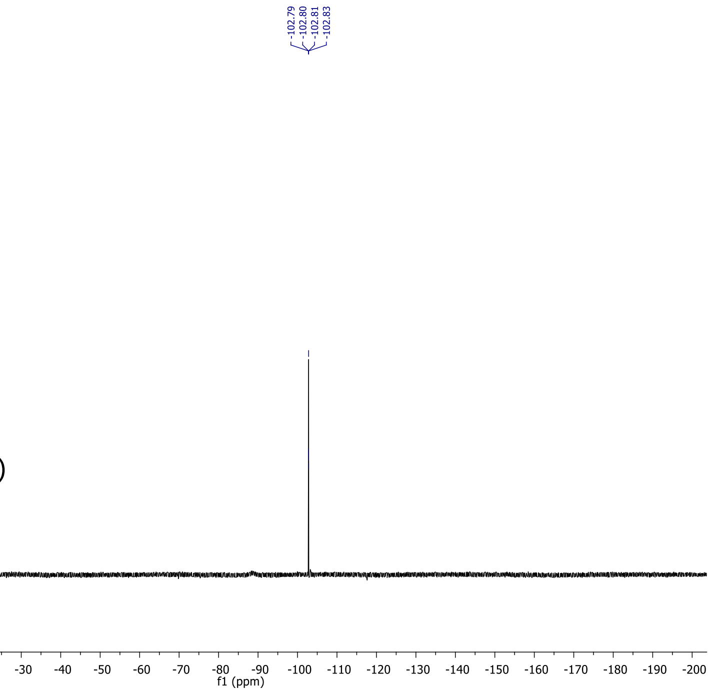

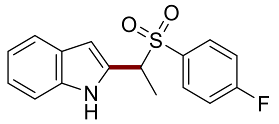

3e

^{19}F NMR (376 MHz, $CDCl_3$)

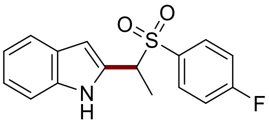

3e

^{13}C NMR (101 MHz, CDCl$_3$)

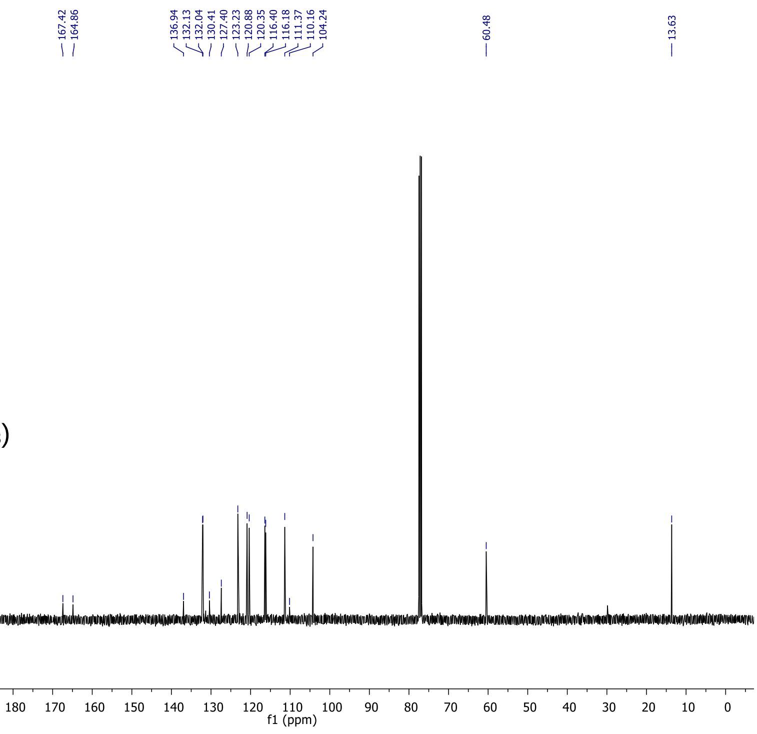

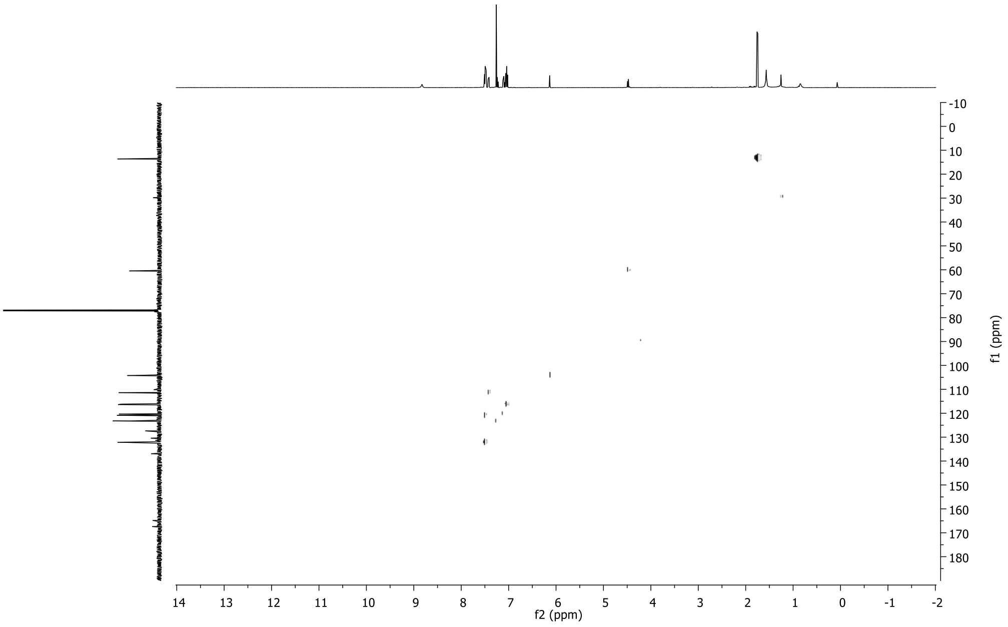

HSQC 2D spectrum of compound **3e** (400 MHz, CDCl$_3$)

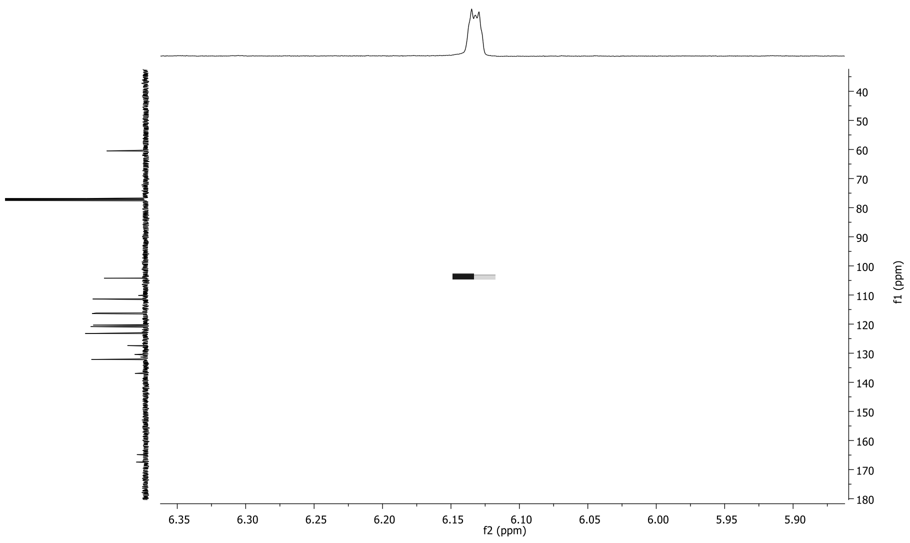

Zoom of HSQC 2D spectrum of compound **3e** (400 MHz, $CDCl_3$)

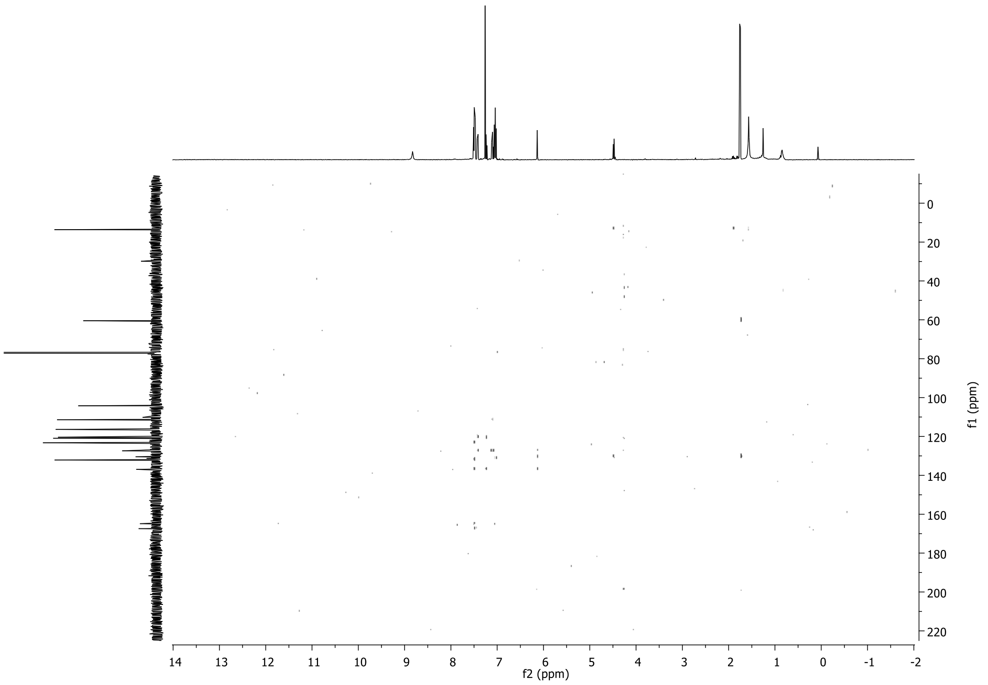

HMBC 2D spectrum of compound **3e** (400 MHz, $CDCl_3$)

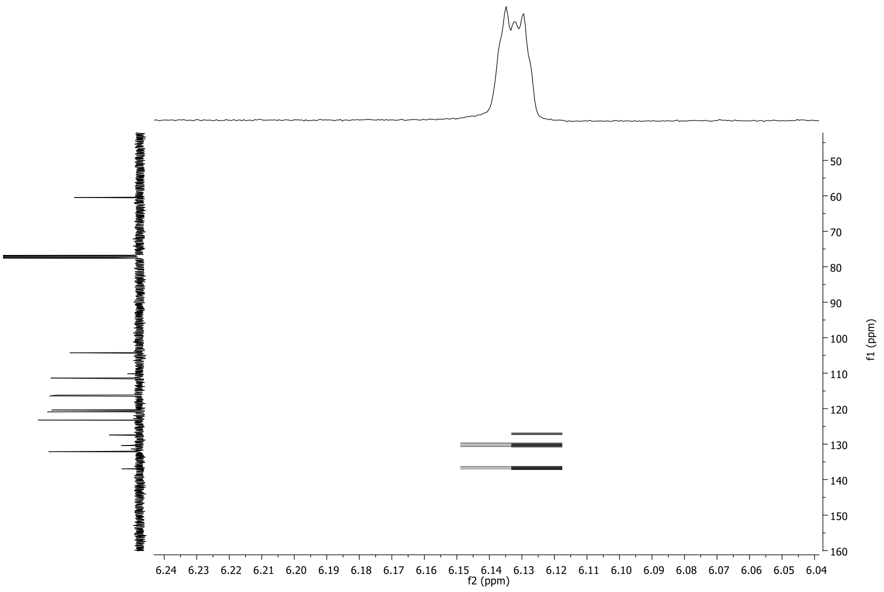

Zoom of HMBC 2D spectrum of compound **3e** (400 MHz, $CDCl_3$)

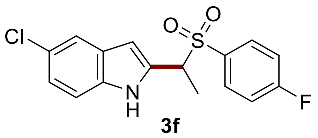

3f

^{1}H NMR (499 MHz, $CDCl_3$)

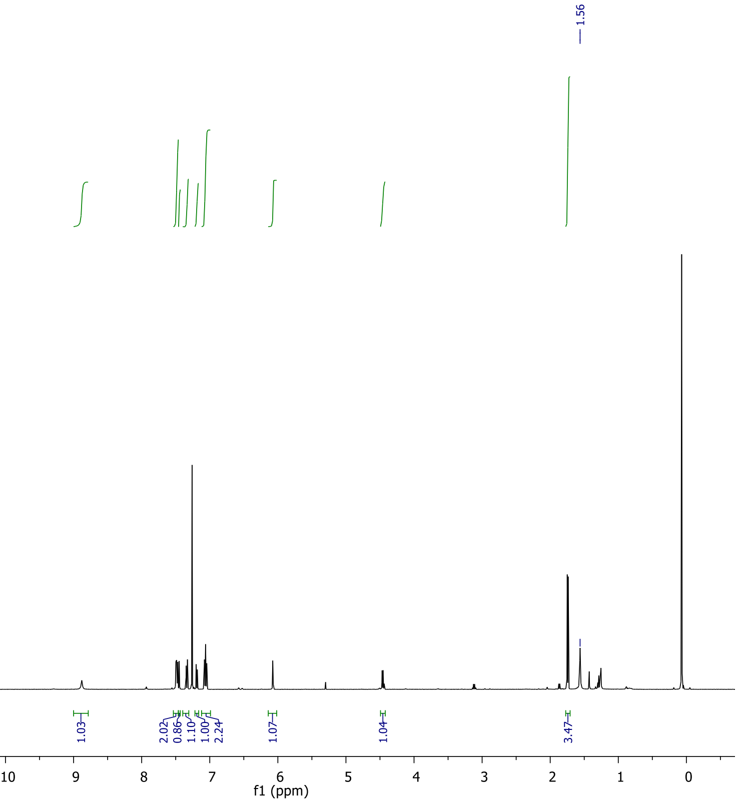

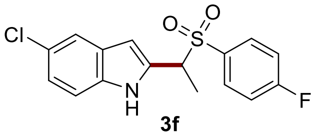

3f

^{19}F NMR (101 MHz, $CDCl_3$)

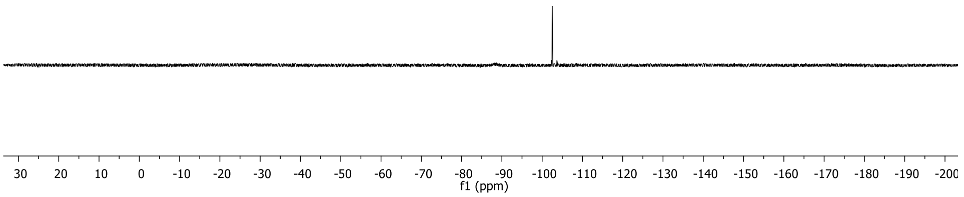

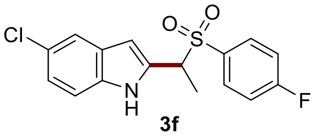

3f

^{13}C NMR (126 MHz, $CDCl_3$)

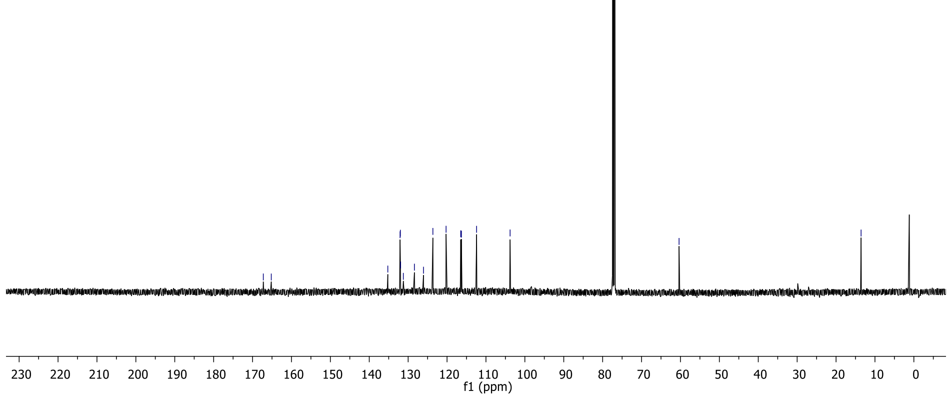

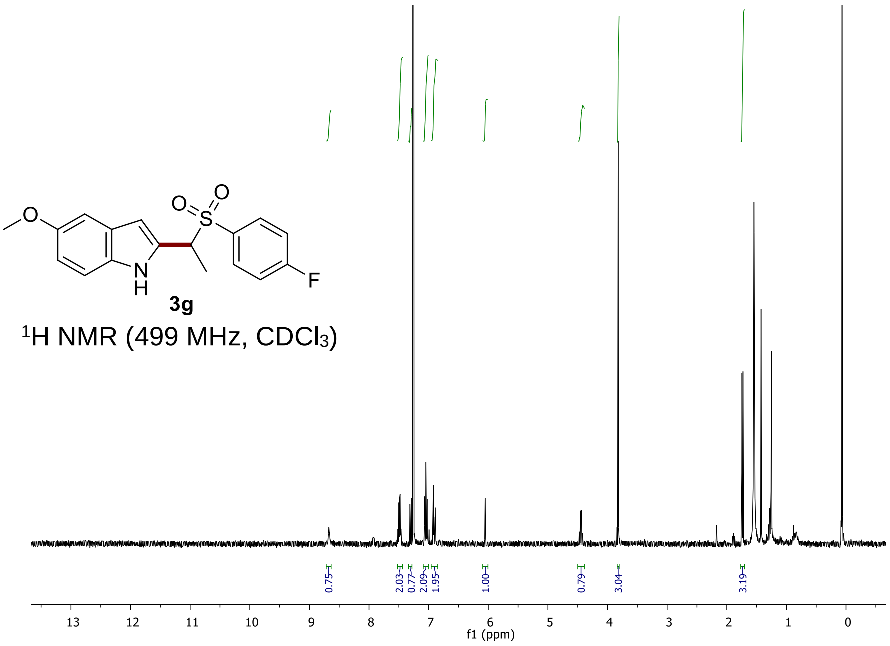

3g

^{1}H NMR (499 MHz, CDCl$_3$)

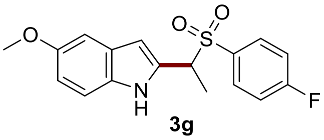

3g

^{19}F NMR (101 MHz, CDCl$_3$)

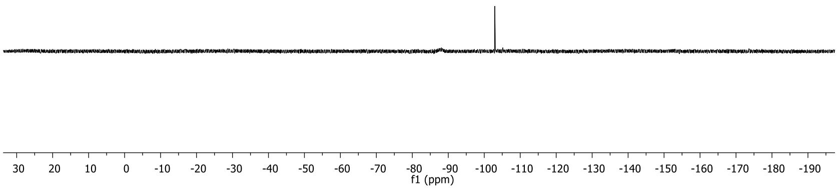

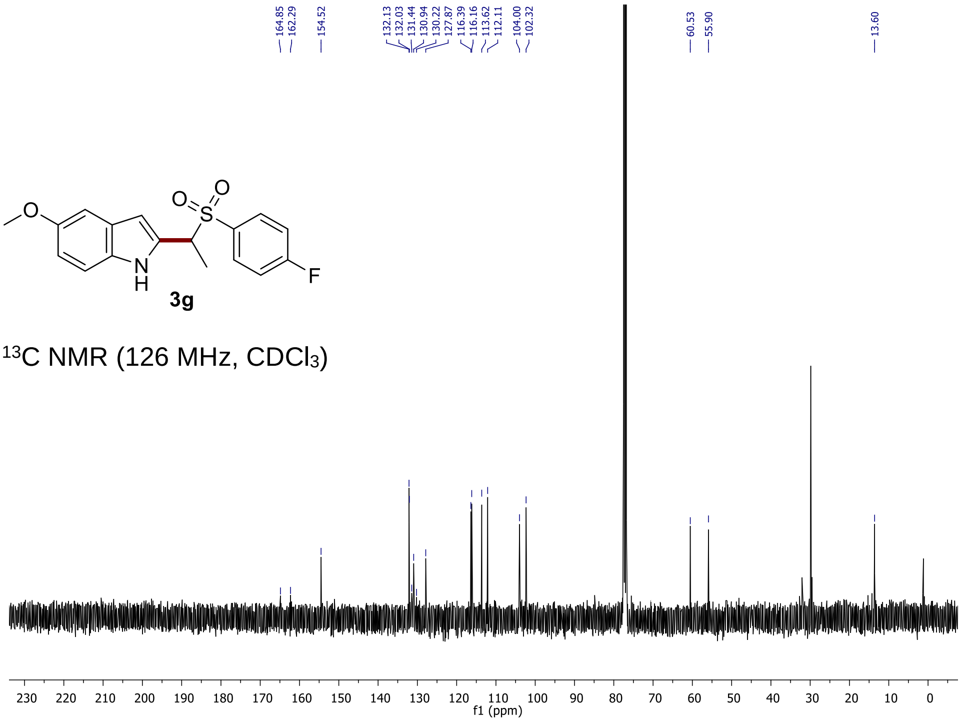

3g

^{13}C NMR (126 MHz, CDCl$_3$)

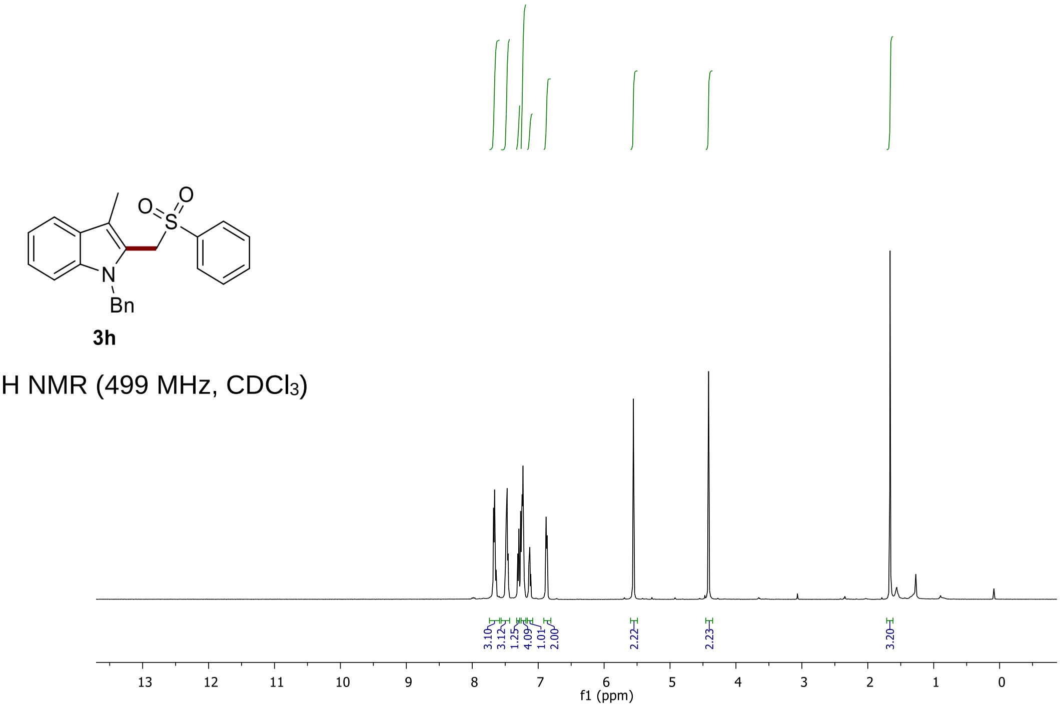

3h

^{1}H NMR (499 MHz, CDCl$_3$)

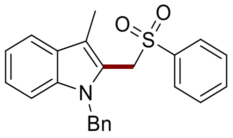

3h

^{13}C NMR (101 MHz, CDCl$_3$)

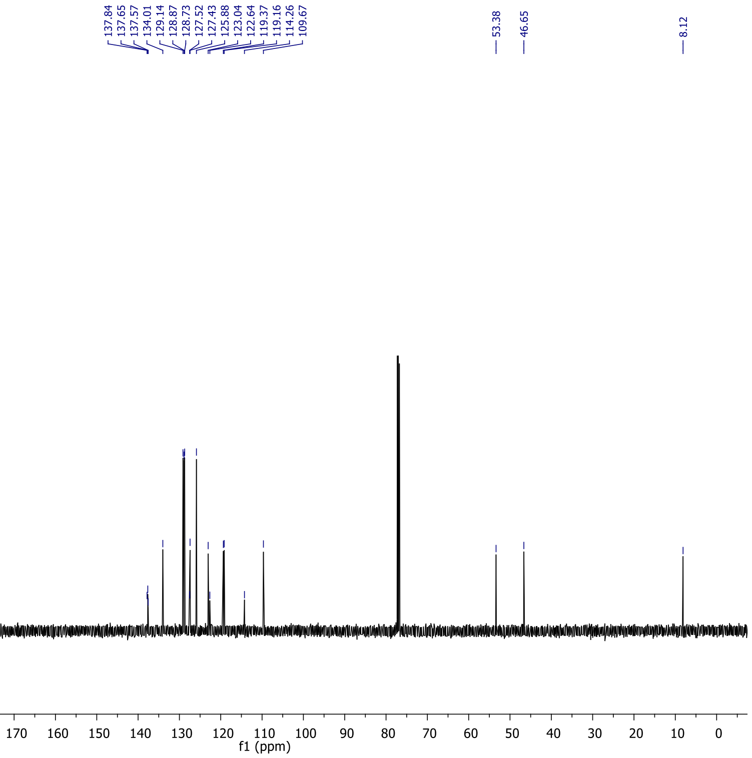

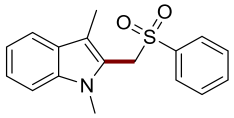

3i

^{1}H NMR (499 MHz, CDCl$_3$)

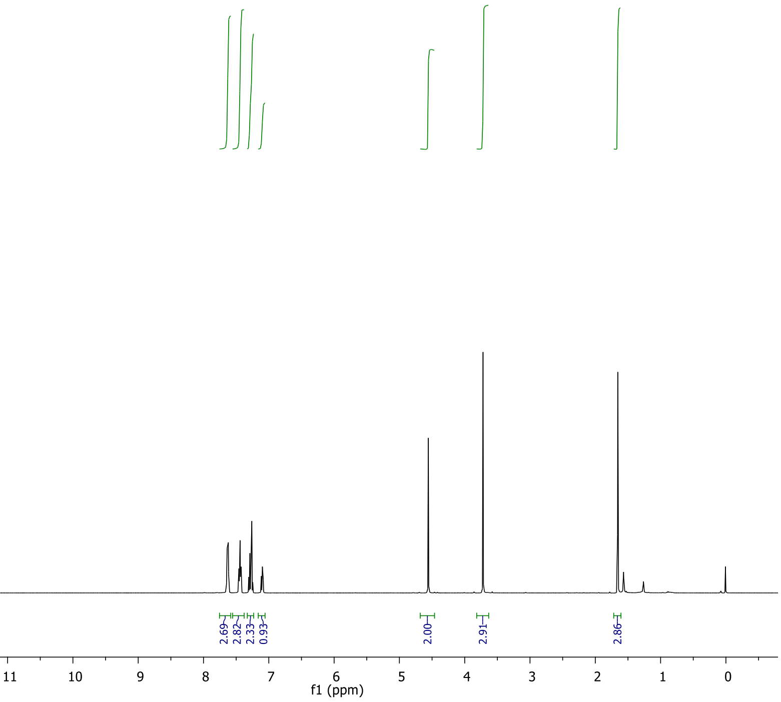

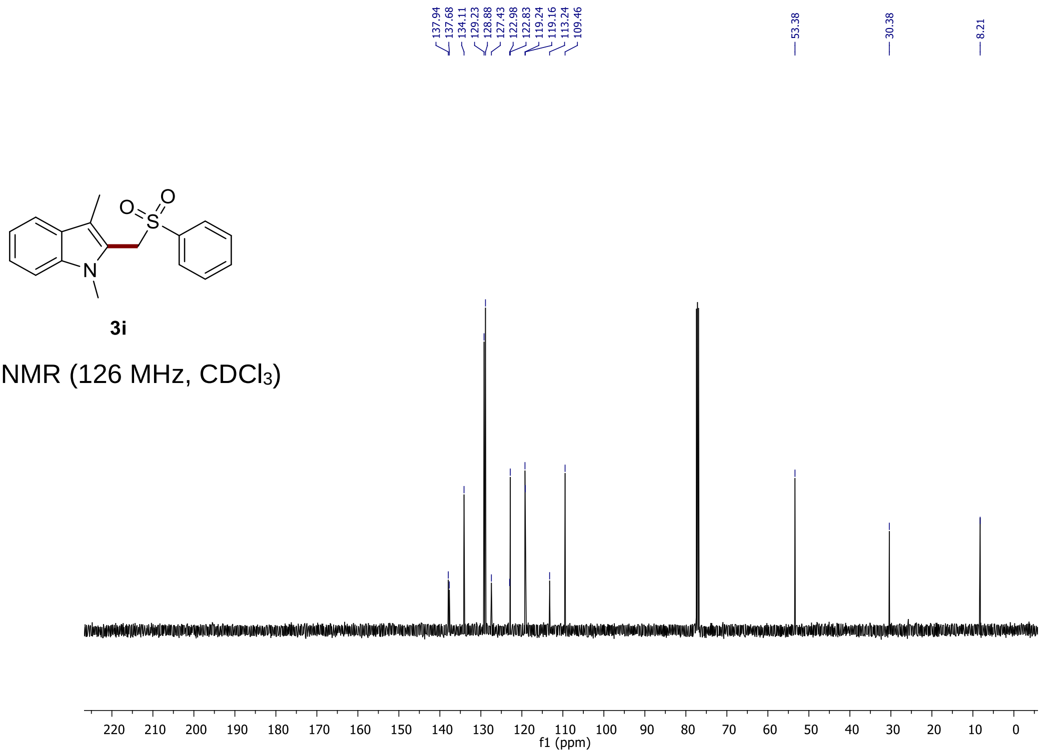

3i

^{13}C NMR (126 MHz, $CDCl_3$)

7. References

(1) Mari, G.; De Crescentini, L.; Favi, G.; Santeusanio, S.; Mantellini, F. *European J. Org. Chem.* **2020**, *2020*, 5411–5424. doi:10.1002/ejoc.202000845

(2) Shao, C.; Shi, G.; Zhang, Y.; Pan, S.; Guan, X. *Org. Lett.* **2015**, *17*, 2652–2655. doi:10.1021/acs.orglett.5b01024

(3) Madabhushi, S.; Jillella, R.; Sriramoju, V.; Singh, R. *Green Chem.* **2014**, *16*, 3125–3131. doi:10.1039/c4gc00246f

(4) Rosso, C.; Cuadros, S.; Barison, G.; Costa, P.; Kurbasic, M.; Bonchio, M.; Prato, M.; Dell'Amico, L.; Filippini, G. *ACS Catal.* **2022**, *12*, 4290–4295. doi:10.1021/acscatal.2c00565

(5) Cuadros, S.; Rosso, C.; Barison, G.; Costa, P.; Kurbasic, M.; Bonchio, M.; Prato, M.; Filippini, G.; Dell'Amico, L. *Org. Lett.* **2022**, *24*, 2961–2966. doi:10.1021/acs.orglett.2c00604

(6) Filippini, G.; Silvi, M.; Melchiorre, P. *Angew. Chemie - Int. Ed.* **2017**, *56*, 4447–4451. doi:10.1002/anie.201612045

(7) Nobile, E.; Castanheiro, T.; Besset, T. *Chem. Commun.* **2021**, *57*, 12337–12340. doi:10.1039/d1cc04737j

(8) Gu, Y.; Leng, X.; Shen, Q. *Nat. Commun.* **2014**, *5*. doi:10.1038/ncomms6405

(9) Prakash, G. K. S.; Hu, J.; Wang, Y.; Olah, G. A. *Org. Lett.* **2004**, *6*, 4315–4317. doi:10.1021/ol048166i

(10) Mi, X.; Kong, Y.; Zhang, J.; Pi, C.; Cui, X. *Chinese Chem. Lett.* **2019**, *30*, 2295–2298. doi:10.1016/j.cclet.2019.09.040
